# Bioorthogonal Engineered Virus-Like Nanoparticles for Efficient Gene Therapy

**DOI:** 10.1007/s40820-023-01153-y

**Published:** 2023-08-12

**Authors:** Chun-Jie Bao, Jia-Lun Duan, Ying Xie, Xin-Ping Feng, Wei Cui, Song-Yue Chen, Pei-Shan Li, Yi-Xuan Liu, Jin-Ling Wang, Gui-Ling Wang, Wan-Liang Lu

**Affiliations:** 1 State Key Laboratory of Natural and Biomimetic Drugs, Beijing Key Laboratory of Molecular Pharmaceutics and Drug Delivery Systems, and School of Pharmaceutical Sciences, Peking University, Beijing, 100191 People’s Republic of China; 2 Jiangsu Collaborative Innovation Center of Chinese Medicinal Resources Industrialization, Nanjing University of Chinese Medicine, Nanjing, 210023 People’s Republic of China; 3 School of Medicine & Holistic Integrative Medicine, Nanjing University of Chinese Medicine, Nanjing, 210023 People’s Republic of China; 4 School of Chemical Sciences, University of Chinese Academy of Sciences, Beijing, 100049 People’s Republic of China

**Keywords:** Virus-like nanoparticle, Site-specific codon mutation, Recombinant biosome, Bioorthogonal chemistry, Gene therapy

## Abstract

Gene therapy offers potentially transformative strategies for major human diseases. However, one of the key challenges in gene therapy is developing an effective strategy that could deliver genes into the specific tissue. Here, we report a novel virus-like nanoparticle, the bioorthgonal engineered virus-like recombinant biosome (reBiosome), for efficient gene therapies of cancer and inflammatory diseases. The mutant virus-like biosome (mBiosome) is first prepared by site-specific codon mutation for displaying 4-azido-L-phenylalanine on vesicular stomatitis virus glycoprotein of eBiosome at a rational site, and the reBiosome is then prepared by clicking weak acid-responsive hydrophilic polymer onto the mBiosome via bioorthogonal chemistry. The results show that the reBiosome exhibits reduced virus-like immunogenicity, prolonged blood circulation time and enhanced gene delivery efficiency to weakly acidic foci (like tumor and arthritic tissue). Furthermore, reBiosome demonstrates robust therapeutic efficacy in breast cancer and arthritis by delivering gene editing and silencing systems, respectively. In conclusion, this study develops a universal, safe and efficient platform for gene therapies for cancer and inflammatory diseases.

**Supplementary Information:**

The online version contains supplementary material available at 10.1007/s40820-023-01153-y.

## Highlights

- A virus-like nanoparticle (reBiosome) was developed via site-specific codon mutation for displaying unnatural amino acid (Azi) on virus envelope protein at a rational site, followed by conjugating weak acid-responsive polyethylene glycol polymer on Azi via bioorthogonal chemistry.
- The reBiosome exhibited reduced virus-like immunogenicity, prolonged blood circulation and enhanced delivery to weakly acidic disease foci.
- The reBiosome enabled efficient delivery of gene editing and gene silencing system, demonstrating remarkable therapeutic efficacy in breast cancer and arthritis, respectively.

**Supplementary Information:**

The online version contains supplementary material available at 10.1007/s40820-023-01153-y.

## Graphical abstract

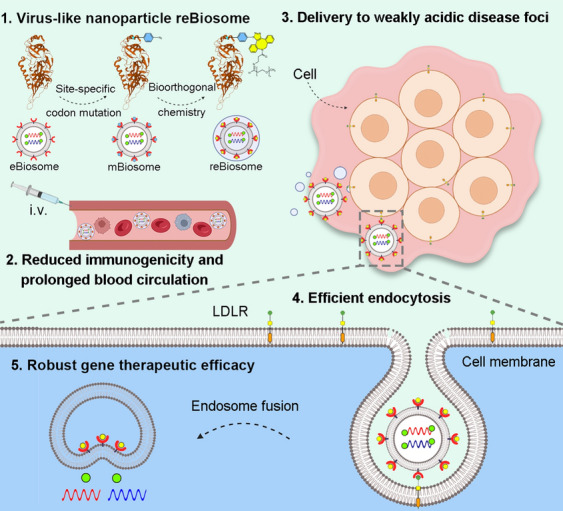

## Introduction

Gene therapy refers to the employment of nucleic acids (DNA or RNA) to treat, cure, or prevent various disorders [1]. Gene therapy could provide a promising treatment strategy for severe diseases that are resistant to traditional treatments. One of the keys to successful gene therapy is to develop an efficient strategy to deliver genes into target tissues with a low adverse effect. So far, scientists have made important achievements in the development of viral vectors that are well tolerated for ex vivo gene therapy and non-viral vectors with high transfection efficiency for in vivo gene therapy [2–6]. Nonetheless, for treating severe diseases via systemic gene therapy, how to design a suitable delivery carrier that could deliver gene into the specific tissue remains a critical scientific issue to be solved.

Virus-like particles (VLPs) are a class of non-viral vectors that possess a structural resemblance to virus, but lack the genetic material required for self-replication [7]. Generally, VLPs consist of capsid proteins, which form a shell that protects gene cargoes in the internal cavity. The assembly and budding of VLPs from host cells are orchestrated by the capsid proteins, which not only confer the structural integrity but also shield the cargo from unfavorable exposures such as extreme pH, temperature, as well as proteolytic and nucleolytic enzymes [8]. Some VLPs also possess an enveloped structure outside of the capsid protein, which consists of a lipid membrane derived from the host and adorned with virus glycoproteins. These glycoproteins play a crucial role in facilitating the attachment of the enveloped VLPs to specific receptors on the host cell membrane, thereby mediating their internalization through the process of endocytosis [9]. Unlike the enveloped VLPs, non-enveloped VLPs rely on the capsid protein itself to mediate entry into the target cells.

Compared to viral vectors, VLPs offer a higher level of safety for systemic gene therapy as they do not present the risk of random integration of virus genes into the human genome or the possibility of massive virus replication. Furthermore, VLPs have extraordinary abilities that have been inherited from billion years of virus evolution. One notable ability of VLPs is that they could self-assemble specific gene cargoes within the host cell by capsid protein. Besides, VLPs could efficiently penetrate endosomes and subsequently release gene cargoes through a variety of in vivo mechanisms, including pore formation on the endosome membrane, low-pH and other events triggering structural conformational changes in glycoproteins and capsid proteins [10–12]. These mechanisms are critical to ensure optimal gene expression. In contrast, non-viral vectors such as liposomes [13], human serum albumin nanoparticles [14], chitosan nanoparticles [15], and manganese dioxide nanoparticles [16] need a delicate design and a complicated in vitro gene packaging process for endowing the nanoparticles with the capabilities of endosome escape and gene expression [17, 18]. So far, several VLP-based vaccines have been approved for clinical use since the highly repetitive arrangement of glycoproteins or capsid proteins on the surface of VLPs can strongly activate the immune system in the body [19, 20]. However, VLPs have not yet been approved for clinical gene therapy. Most likely, this is due to the fact that VLPs can trigger strong but unwanted virus-like proinflammatory reactions, leading to rapid clearance by the immune system and a low gene delivery efficiency at disease tissues such as tumor or arthritis tissue.

**Fig. 1:**
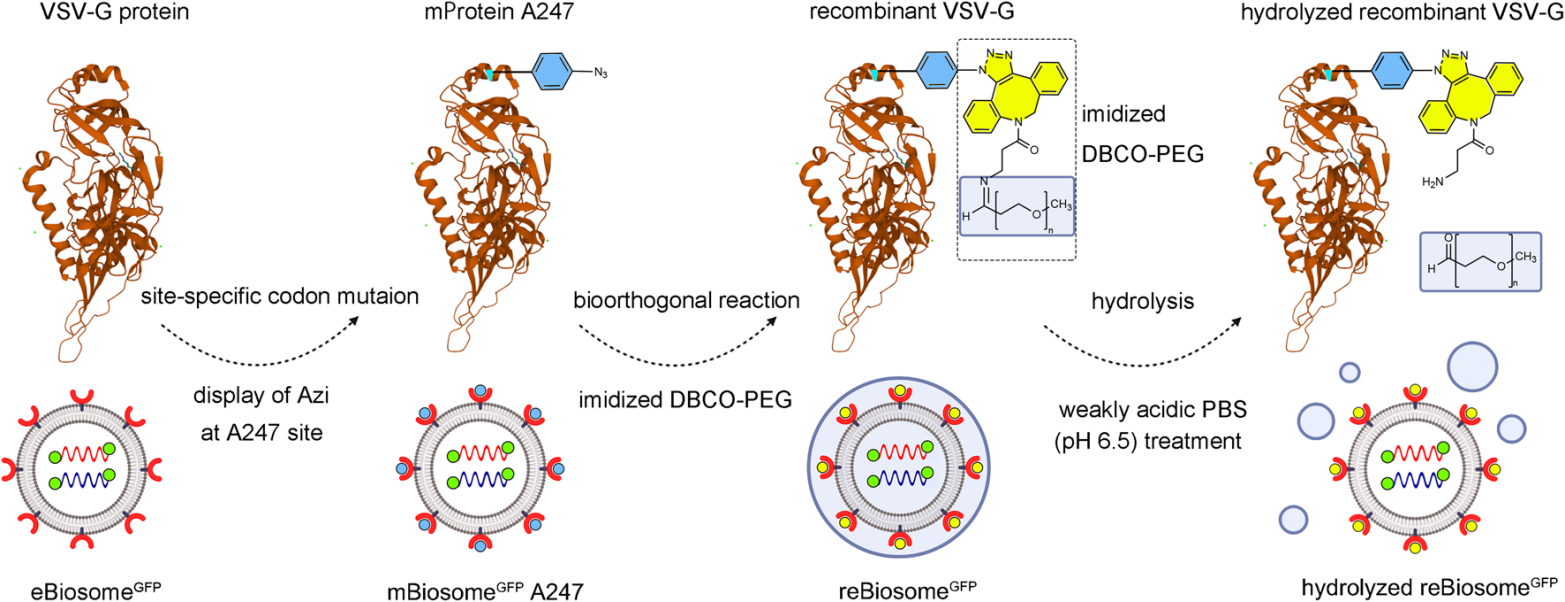
Illustrations for the design of reBiosome and its pH responsiveness. The mutated VSV-G enveloped biosome (mBiosome) is developed by site-specific codon mutation for displaying Azi (4-azido-L-phenylalanine, Azi; a type of unnatural amino acid) on the VSV-G enveloped biosome (eBiosome). The recombinant biosome (reBiosome) is further developed by incorporating the imidized hydrophilic polymer (imidized DBCO-PEG) onto the Azi of mBiosome via bioorthogonal chemistry. When treated with weak acid, the imidized DBCO-PEG on the reBiosome is hydrolyzed, and the transfection capability of reBiosome could be recovered. VSV-G represents the vesicular stomatitis virus glycoprotein, a kind of virus envelope protein; imidized DBCO-PEG represents dibenzocyclooctyne-polyethylene glycol 2000. **Top:** The red ribbon downloaded from PBD database (https://doi.org/10.2210/pdb5OYL/pdb) indicates VSV-G. **Bottom:** The lipid bilayer in gray indicates the envelope. The red bowl indicates the envelope protein VSV-G. The green circle indicates the capsid protein. The blue dot indicates the Azi. The spiral structure indicates the gene cargoes packaged (GFP mRNA for example). The bluish circle indicates the imidized DBCO-PEG. (Color figure online)

In the study, we rationally designed and bioorthogonal engineered a recombinant virus-like nanoparticle (named reBiosome) with the aim to reduce the immunogenicity of viral proteins and realize efficient gene therapy for cancer and inflammatory diseases. In the structure, the glycoprotein of vesicular stomatitis virus (VSV-G) was used as the viral envelope protein since it is widely employed in pseudotyping viruses due to its resistance to mechanical force [21]. Besides, the paternally expressing protein 10 (capsid 10) was used as the viral capsid protein as it is an endogenous capsid protein in mammalian cells and could package, secrete, and deliver specific RNAs [22]. On the basis of these two proteins, we constructed the VSV-G enveloped nanoparticle, named eBiosome. Furthermore, the reBiosome was developed by site-specific codon mutation for displaying 4-azido-L-phenylalanine (Azi; a type of unnatural amino acid) on VSV-G of eBiosome at a rational site, followed by incorporating weak acid-responsive polyethylene glycol (PEG) polymers on the envelope via bioorthogonal chemistry (Fig. 1). In this design, the codon mutation site for the display of Azi was carefully selected to reserve the high cellular endocytosis capacity of VSV-G. Bioorthogonal chemistry was employed to click the weak acid-responsive PEG polymer to VSV-G with high efficiency in the physical condition. These two techniques endow reBiosome with reduced immunogenicity, prolonged blood circulation, and enhanced gene delivery efficiency. The reduced immunogenicity of reBiosome was verified by testing the levels of granulocytes, monocytes, and virus-like pro-inflammatory cytokines in the blood of mice. The prolonged blood circulation of reBiosome was verified by testing the content in the blood of mice at various time points. The high delivery efficiency of reBiosome to weakly acidic foci was verified by the expression of target genes in tumor tissues from breast cancer-bearing mice and in arthritic tissues from collagen-induced arthritis mice. At last, the potential of reBiosome in gene therapy was explored by packaging a gene editing system in treating breast cancer-bearing mice, and by packaging a gene silencing system in treating collagen-induced arthritis mice, respectively.

## Experimental Section

LC-MS/MS and Western blot were employed to validate the specific display of Azi to VSV-G and the expression of mutated VSV-G, respectively. Transmission electron microscopy and dynamic light scattering were employed to analyze the morphology, the size and the zeta potentials for biosomes, respectively. Surface plasmon resonance, ultrahigh-resolution confocal laser scanning microscopy and flow cytometry were employed to analyze the affinity of biosomes to LDLR (low-density lipoprotein receptor), the co-localization of biosomes with LDLR, and the transfection efficiency of biosomes, respectively. Fluorescence imaging was employed to study the pharmacokinetic and distribution of biosomes in mice. Detailed material and methods descriptions can be found in the Supporting Information.

## Results and Discussion

### Site-Specific Codon Mutated Virus-Like mBiosome and Its Gene Transfection Efficiency

**Fig. 2:**
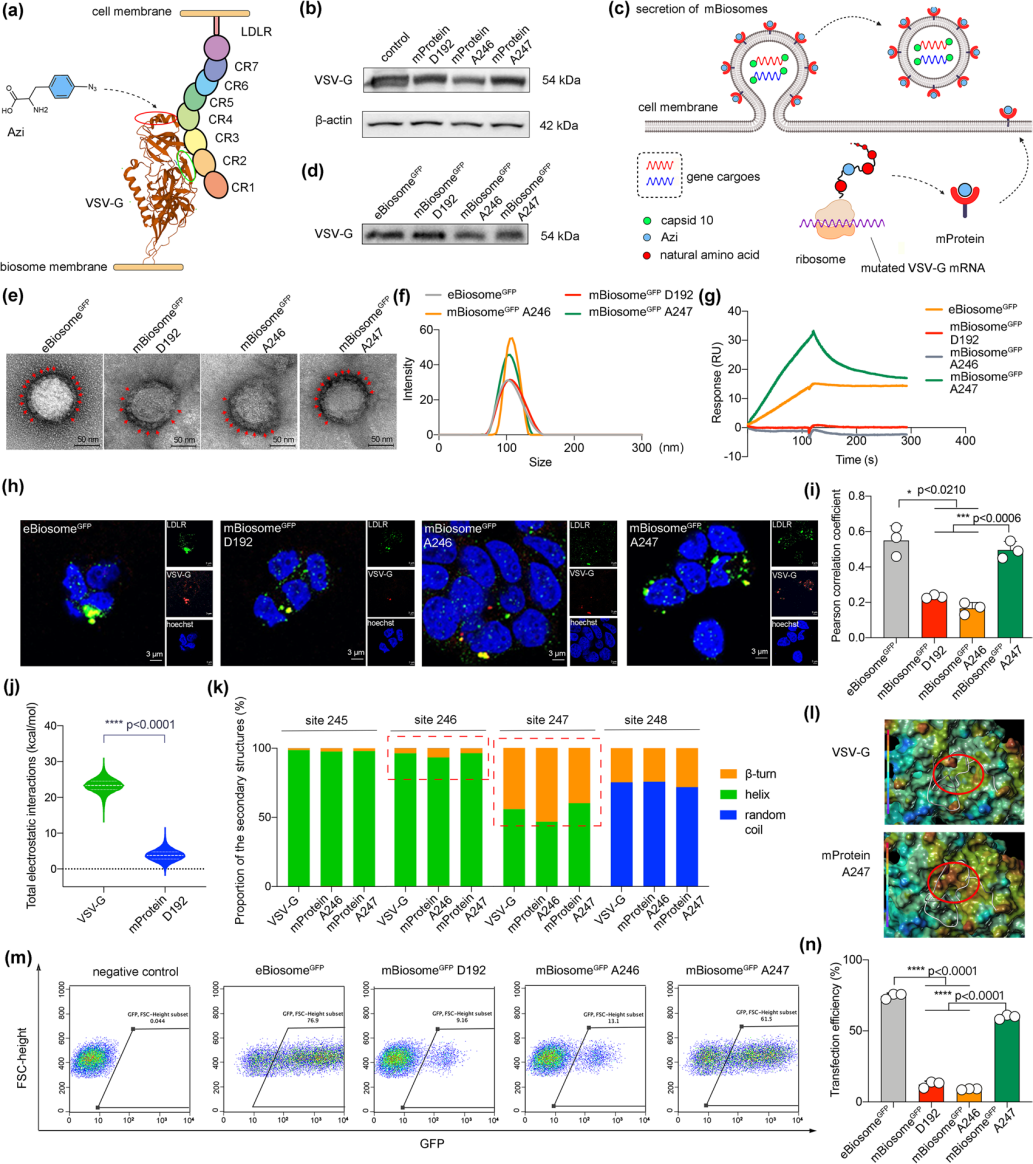
Virus-like mBiosome by site-specific display of an unnatural amino acid and its gene transfection efficiency. **a** Site selection for the display of Azi (a type of unnatural amino acid) on VSV-G. The interaction between LDLR and VSV-G is illustrated using ribbon representations created by Mol software. The green circle highlights the domain of VSV-G that interacts with LDLR CR2, while the red circle indicates the chosen locations for Azi display. **b** Expression of mutated VSV-G proteins in cells. The expression of mutated VSV-G proteins (mProteins) was detected by Western blot, respectively. β-actin was used as the internal reference. **c** Illustration for the display process of Azi on mProtein and the secretion process of mBiosome^GFP^. VSV-G is displayed with Azi (blue circle) at the ribosome, followed by folding and transporting to the cell membrane. **d** Incorporation of VSV-G in mBiosomes^GFP^. The membrane proteins of mBiosomes were extracted and analyzed by Western blot. **e** TEM images for mBiosomes^GFP^. Red arrows indicate the spikes. The scale bar represents 50 nm. **f** Particle size distributions of mBiosomes^GFP^. The study was performed by dynamic light scattering (DLS). **g** Binding curves of varying mBiosomes^GFP^ with LDLR. The study was conducted by surface plasma resonance (SPR). **h** Representative co-localization images of mBiosomes^GFP^ with LDLR captured by CLMS. Red: VSV-G; green: LDLR; blue: nucleus. The scale bar indicates 3 μm. **i** Pearson's correlation coefficient of the VSV-G and LDLR based on the images of Fig. 2h. **j** Total electrostatic interactions in the surrounding residues of 192 site. **k** Secondary structures of residues between 245 and 247 sites. The proportion % of the secondary structures, including *β*-turn, helix, and random coil at surrounding sites (sites 245, 246, 247 and 248) in unmutated VSV-G, mProtein A246 and mProtein A247. **l** Electrostatic potential maps in the joint surface between VSV-G and LDLR. Red circles, the joint surface; gray bands, the LDLR CR2 motif. The map's color from red to purple refers to the electrostatic potential from positive to negative. **m** Flow cytometry scatter plots. **n** Transfection efficiency based on GFP expression. Data are represented as the mean ± standard deviation (*n* = 3). The two-tailed unpaired Student's t-test was used to determine the significance between groups. (Color figure online)

It is reported that the VSV-G protein binds to the cysteine-rich repeat (CR) domain of the low-density lipoprotein receptor (LDLR) family on the cell surface and enters the cell efficiently via clathrin-mediated endocytosis pathway [23–25]. Consequently, in the development of mutated VSV-G enveloped biosomes (mBiosomes), it is crucial to carefully select the site for displaying Azi to reserve the high cellular endocytosis capacity of VSV-G. Three residue sites (D192, A246, and A247) on the top domain (red circle) of VSV-G, but distal to the joint surface (green circle) to LDLR CR2, were chosen for condon mutation and subsequent Azi display (Fig. 2a). The mutated plasmids (pCMV-VSV-G-D192, pCMV-VSV-G-A246 and pCMV-VSV-G-A247) encoding the mutated VSV-G proteins were prepared by introducing the amber codon TAG to the respective selected sites (D192, A246, and A247) in the pCMV-VSV-G plasmid. These mutations were confirmed through DNA sequencing (Fig. S1). Subsequently, the mutated pCMV-VSV-G plasmid and the orthogonal aminoacyl tRNA synthetase/tRNACUA pair-expressing plasmid (pIRE4-Azi) were co-transfected into HEK 293 T cells, supplemented with 1 mM Azi, leading to the site-specific display of Azi and the generation of the mutated VSV-G proteins (mProtein D192, mProtein A246, and mProtein A247). These outcomes were verified using liquid chromatography with tandem mass spectrometry (LC-MS/MS) (Fig. S2) and Western blot (Fig. 2b), respectively.

To prepare the mutated VSV-G enveloped biosome packaging the GFP mRNA (mBiosome^GFP^), a series of steps were undertaken. Firstly, HEK 293T cells were co-transfected with the mutated pCMV-VSV-G plasmid along with other three plasmids, pIRE4-Azi, pCMV-capsid10-flag (encoding the capsid10 protein), and pCMV-cargoGFP (encoding the GFP mRNA). This transfection process was carried out in the presence of 1 mM Azi, as depicted in Fig. 2c. After the concentration of mBiosomes, the membrane proteins were extracted and analyzed by Western blot assay. The results clearly demonstrated a successful incorporation of mutated VSV-G proteins into the envelope of mBiosomes (Fig. 2d). In order to gain further insights, the morphologies, particle sizes and Zeta potentials of the mBiosomes were examined using transmission electron microscopy (TEM) and dynamic light scattering (DLS). The TEM images showed that eBiosome had a spherical shape and was encapsulated with spike proteins, closely resembling general enveloped virus. Similarly, three different types of mBiosomes displayed similar morphological characteristics to the eBiosome (Fig. 2e). Moreover, the particle sizes and Zeta potential values of the three different types of mBiosomes^GFP^ were evaluated and compared to the eBiosome^GFP^. The results obtained from DLS analysis demonstrated that all three types of mBiosomes^GFP^ shared comparable particle sizes and Zeta potential values with the eBiosome^GFP^ (Figs. 2f and S3).

To determine the binding affinity between mBiosome^GFP^ and LDLR, the formulations of eBiosome^GFP^, mBiosome^GFP^ D192, mBiosome^GFP^ A246, and mBiosome^GFP^ A247 were passed through the LDLR immobilized chip for surface plasmon resonance (SPR) assay. The results showed that the eBiosome^GFP^ and mBiosome A247 displayed a rapid and strong binding with LDLR. Compared to the eBiosome^GFP^, the mBiosome A247 bound to LDLR and dissociated from LDLR in a quicker manner. In contrast, mBiosome A246 and mBiosome D192 exhibited a reduced affinity to LDLR (Fig. 2g). To further validate the binding affinity between mBiosome^GFP^ and LDLR, the formulations of eBiosome^GFP^, mBiosome^GFP^ D192, mBiosome^GFP^ A246, and mBiosome^GFP^ A247 were incubated with cells, and the co-localization of VSV-G (red) with LDLR (green) was studied by immunofluorescence staining (Fig. 2h). Consistent with the SPR results, the co-localization of mBiosome^GFP^ A247 with LDLR was significantly higher than that of mBiosome^GFP^ A246 or mBiosome^GFP^ D192 (Fig. 2i).

To gain a deep understanding of the underlying mechanism, we conducted molecular dynamic simulations to investigate the properties of different VSV-G proteins in the pre-fusion conformation. The original amino acid residue at site 192 was aspartic acid (abbreviated as D in this study, Fig. S4a), which carried a negative electricity in a neutral environment and engaged in electrostatic interactions with surrounding residues. Our simulations revealed that the electrostatic interactions among the surrounding residues at site 192 in VSV-G were significantly stronger than those observed in mProtein D192 (Fig. 2i). Since strong electrostatic interactions contribute to the stability of protein structures [26, 27], it is likely that mProtein D192 may not be stable in the pre-fusion conformation.

Furthermore, we examined the effects of introducing Azi to sites 246 and 247 on the secondary structures. The results showed that the amino acid residues at sites 246 and 247 were located within the same terminal region of the helical secondary structure (Fig. S4b). Interestingly, the introduction of Azi to site 246 caused evident changes in the secondary structures of the surrounding residues in mProtein A246 (red rectangles, Fig. 2j). In contrast, the introduction of Azi to site 247 had minimal impact on the original secondary structures in mProtein A247 (Fig. 2j). These findings suggest that the display of Azi at sites 192 or 246 destabilizes the conformation of VSV-G, leading to the reduced binding affinities of mBiosome D192 and mBiosome A246 to LDLR. Moreover, we observed an increase in the electrostatic potential density at the joint surface of mProtein A247 compared to that of VSV-G (Fig. 2k). Previous reports have demonstrated that electrostatic potential can enhance binding rates in protein–protein complexes [28, 29], showing that the increased electrostatic potential density at the joint interface could accelerate the binding process between mProtein A247 and LDLR, as well as the dissociation process of mProtein A247 from LDLR.

Upon internalization via LDLR-mediated endocytosis, the VSV-G protein undergoes a conformational change from its pre-fusion state to a post-fusion conformation, triggered by the acidic environment in the endosome. This conformational transition disrupts the receptor-binding sites and exposes the membrane fusion domain. Consequently, the VSV-G enveloped biosome fuses with the endosomal membrane, leading to the release of gene cargoes and realizing high gene transfection efficiency [30, 31]. To assess the gene transfection efficiency, cells were treated with different variants of mBiosomes^GFP^, and the expression of GFP protein was quantified using flow cytometry (Fig. 2m). The proportion of GFP-positive cells was calculated to determine the transfection efficiency. The results clearly indicated that mBiosome^GFP^ A247 had the highest transfection efficiency, reaching up to 60%. This efficiency was significantly superior to that of mBiosome^GFP^ D192 or mBiosome^GFP^ A246 (Fig. 2n). These findings collectively indicate that mBiosome^GFP^ A247 had the strongest affinity to the receptor and achieved the highest transfection efficiency among the three different types of mBiosomes.

### Bioorthogonal Engineered Virus-Like reBiosome and Its pH Responsiveness

**Fig. 3:**
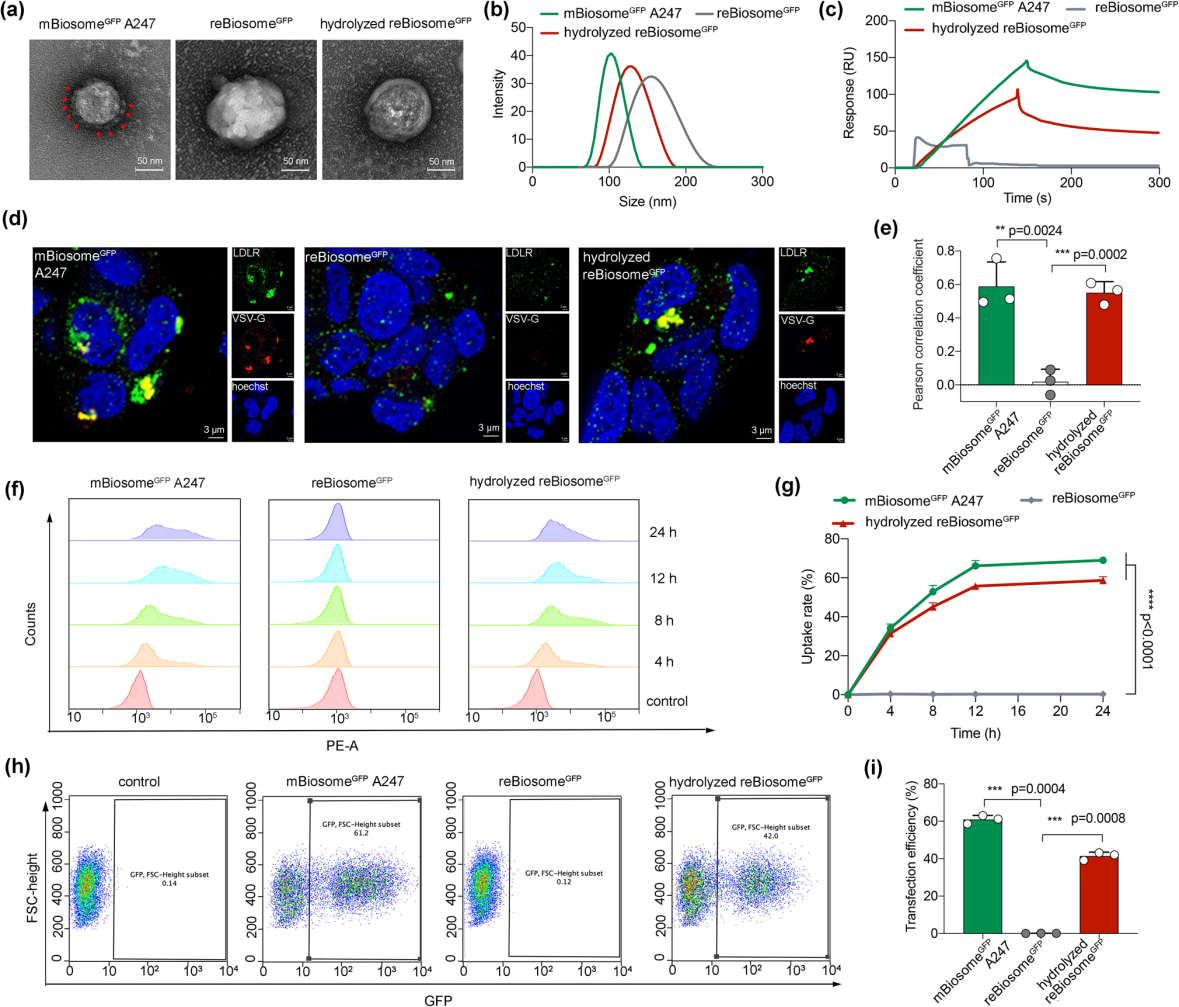
Bioorthogonal engineered virus-like reBiosome by incorporating stealth material and its pH responsiveness. **a** TEM images of reBiosome^GFP^ and hydrolyzed reBiosome^GFP^. Red arrows indicate spikes. The scale bar represents 50 nm. **b** Size distributions of reBiosome^GFP^ and hydrolyzed reBiosome^GFP^. The study was performed by DLS. **c** Specific binding of hydrolyzed reBiosome^GFP^ with LDLR. The study was performed by SPR. **d** Representative co-localization pictures of hydrolyzed reBiosome^GFP^ with LDLR captured by CLMS. Red: VSV-G; green: LDLR; blue: nucleus. The scale bar represents 3 μm. **e** Pearson's correlation coefficient between VSV-G and LDLR based on Fig. 3d. **f** Flow cytometry histograms of DiI intensity. **g** Time-dependent uptake of hydrolyzed reBiosome^GFP^ based on DiI intensity. **h** Flow cytometry scatter plots. **i** Transfection efficiency based on GFP expression. Data are represented as the mean ± standard deviation (*n* = 3). The two-tailed unpaired Student's t-test was used to determine the significance between groups. (Color figure online)

To prepare reBiosome^GFP^, the mBiosome^GFP^ A247 was reacted with imidized PEG synthesized in-house in PBS (pH 7.4) (details in Supplementary Results). The imidized PEG contained a bioorthogonal reactive group DBCO and a weak acid-responsive group imine. Through the bioorthogonal reaction, the imidized PEG was linked to the azido group on mProtein A247 of reBiosome^GFP^. To confirm the incorporation of the polymer, Western blot analysis was employed and the result showed an increased molecular weight of VSV-G on reBiosome^GFP^ compared to that on mBiosome^GFP^ A247 (Fig. S5). Furthermore, the TEM images showed that reBiosome^GFP^ was more stereoscopic compared to mBiosome^GFP^ A247 (Fig. 3a). Meanwhile, the spike proteins (indicated by red arrows) on mBiosome^GFP^ A247 were not visible in the reBiosome^GFP^. In addition, the results from DLS results revealed that reBiosome^GFP^ had a larger size and a smaller zeta potential value than mBiosome^GFP^ A247 (Figs. 3b and S6). These results indicate a successful preparation of reBiosome^GFP^.

To investigate the hydrolysis of reBiosome^GFP^ in response to weak acid, reBiosome^GFP^ was treated with a weakly acidic medium (PBS pH 6.5). The results from Western blot analysis showed that the molecular weight of VSV-G on the treated reBiosome^GFP^ was decreased as compared to that on the untreated reBiosome^GFP^ (Fig. S5). Moreover, the TEM images showed that the coating layer of reBiosome^GFP^ was hydrolyzed and partially fallen off after treating with weakly acidic PBS (pH 6.5) (Fig. 3a). Moreover, the DLS results indicated a reduction in particle size and less negative Zeta potential of the treated reBiosome^GFP^ compared to the untreated reBiosome^GFP^ (Figs. 3b and S6). These observations suggest that the PEG polymer on the surface of reBiosome^GFP^ could be partially hydrolyzed by weakly acidic PBS (pH 6.5) treatment.

To determine the binding affinity between reBiosome^GFP^ and LDLR, the formulations of reBiosome^GFP^ and hydrolyzed reBiosome^GFP^ were passed through the LDLR immobilized chip for the SPR assay. The results showed that unhydrolyzed reBiosome^GFP^ displayed weak binding with LDLR. In contrast, the hydrolyzed reBiosome^GFP^ exhibited rapid and strong binding with LDLR, showing a similar binding effect to that of mBiosome^GFP^ A247 (Fig. 3c). Besides, CLMS analysis showed that unhydrolyzed reBiosome^GFP^ had little co-localization with LDLR, while hydrolyzed reBiosome^GFP^ had strong co-localization with LDLR (Fig. 3d, e). Furthermore, the uptake rate of hydrolyzed reBiosome^GFP^ was significantly higher than that of unhydrolyzed reBiosome^GFP^ (Fig. 3f, g). In addition, the results from the transfection study indicated that the transfection capability of unhydrolyzed reBiosome^GFP^ was abolished by PEG polymer incorporation while restored by hydrolysis in response to weakly acidic medium treatment (Fig. 3h, i). These observations could be attributed to the fact that the imidized PEG coating layer prevents the binding and the cellular uptake of reBiosome^GFP^ to cells, while the uncoating the imidized PEG coating layer can restore the virus-like high transfection capability of reBiosome^GFP^.

### Reduced Immunogenicity and Prolonged Blood Circulation Time of Virus-Like reBiosome in Mice

**Fig. 4:**
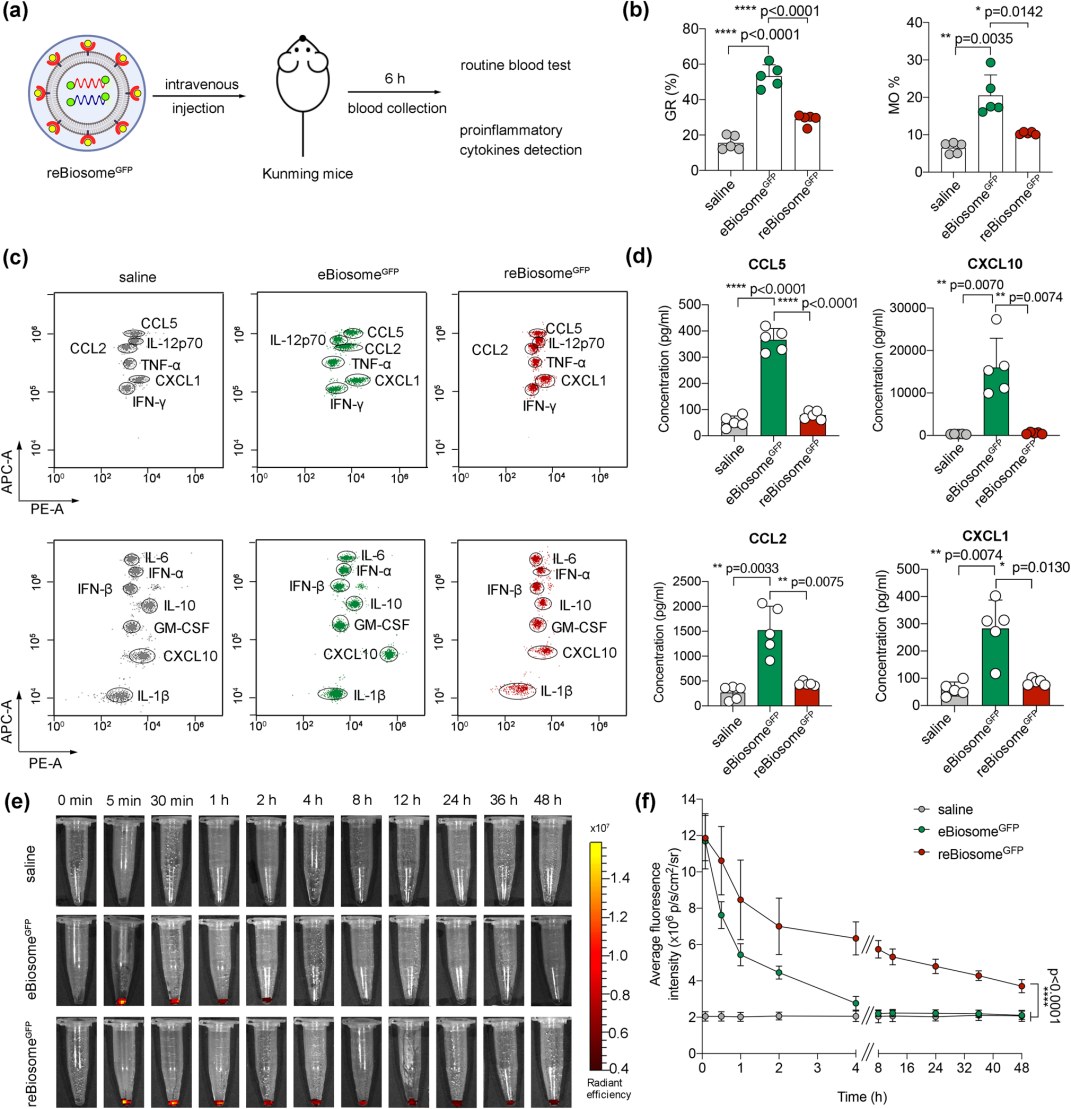
Reduced immunogenicity and prolonged blood circulation time of the virus-like reBiosome in mice. **a** The illustration for immunogenicity detection in vivo. The healthy Kunming (KM) mice were treated with reBiosome^GFP^, eBiosomes^GFP^, and saline (PBS pH 7.4) by intravenous injection, respectively. After 6 h, the blood specimens were collected from each mouse for routine blood tests and proinflammatory cytokine detections. **b** Phagocyte ratios in blood circulation. The Phagocyte ratios were measured by a hematology analyzer, including granulocyte ratio (GR%) and monocyte ratio (MO%) in blood specimens. **c** Flow cytometry scatter plots. **d** Concentrations of chemokines (CCL5, CXCL10, CCL2, and CXCL1) in blood specimens. **e** DiR fluorescence intensity in the blood specimens at varying time points. The KM mice were treated with DiR-labeled reBiosome^GFP^, eBiosomes^GFP^, and saline (PBS pH 7.4) by intravenous injection, respectively. The blood specimens were collected at 5 min, 30 min, 30 min, 1 h, 2 h, 4 h, 8 h, 12 h, 24 h, 36 h, and 48 h, followed by DiR fluorescence imaging, respectively. **f** Pharmacokinetic profiles. The DiR fluorescence intensities of the blood specimens in Fig. 4e were quantified by LivingImage software. Data are represented as the mean ± standard deviation (*n* = 5). In Fig. 4f, the two-way analysis of variance (ANOVA) was used to determine the significance among groups. In other panels, the two-tailed unpaired Student's t-test was used to determine the significance between groups. (Color figure online)

Given the potential of VSV-G to be recognized by the innate immune system in the bloodstream [32], we first assessed the immunogenicity of eBiosome and reBiosome by analyzing the ratios of granulocytes and monocytes in the blood of normal Kunming (KM) mice. These mice were treated with VSV-G enveloped biosome^GFP^ (eBiosome^GFP^) or recombinant biosome^GFP^ (reBiosome^GFP^) via the tail vein, while saline (PBS pH 7.4) was used as the blank control. The blood specimens were collected at 6 h after injection (Fig. 4a). The results obtained from routine blood tests revealed that eBiosome^GFP^ induced an increase in the ratios of granulocytes (GR%) and monocytes (MO%), both of which were major phagocytes to viral infection. In contrast, the ratios of GR% and MO% in reBiosome^GFP^-treated mice were lower compared to those in eBiosome^GFP^-treated mice (Fig. 4b). Furthermore, given that virus-like eBiosome shares several characteristics with virus, it may trigger an immune response in the blood similar to that induced by virus. To verify the reduced immunogenicity of reBiosome compared to eBiosome in the blood, we performed an assessment using a comprehensive anti-virus response panel, consisting of chemokines (CCL5, CXCL10, CCL2, and CXCL1) that are involved in the chemotaxis of granulocytes and monocytes [33–36], interferons (IFN-*α*, IFN-*β*, IFN-*γ*) that are crucial cytokines secreted by host cells in response to viral infections [37], interleukins (IL-1*β*, IL-6, IL-10, IL-12) that play various roles in proinflammation and immunoregulation during virus infection [38–40], tumor necrosis factor-alpha (TNF-*α*) that plays a central role in the control of viral replication [41], as well as GM-CSF that plays a role in promoting immune cell proliferation and enhancing immunity in response to virus infection [42]. Flow cytometry results demonstrated that the levels of chemokines (CCL5, CXCL10, CCL2, and CXCL1) in eBiosome^GFP^-treated mice were higher compared to those in saline-treated mice. In contrast, the levels of these cytokines were significantly lower in reBiosome^GFP^-treated mice compared to eBiosome^GFP^-treated mice (Fig. 4c, d). The levels of other cytokines, including interferons (IFN-*α*, IFN-*β*, IFN-*γ*), interleukins (IL-1*β*, IL-6, IL-10, IL-12), TNF-*α*, and GM-CSF, remained unchanged in all mice treated with the formulations (Fig. S7). These findings indicate that reBiosome exhibits a reduced immunogenicity compared to eBiosome.

To investigate the pharmacokinetics, the formulations of reBiosome^GFP^ and eBiosome^GFP^ were labeled with a lipophilic, near-infrared fluorescent cyanine dye (DiR), followed by intravenously injection into the healthy KM mice. Saline (PBS pH 7.4) was used as the blank control. After dosing, the blood specimens were collected at various time-points (5 min, 15 min, 30 min, 1 h, 2 h, 4 h, 8 h, 12 h, 24 h, 36 h, and 48 h), and observed by DiR fluorescence imaging. The results from the fluorescence intensity-time profile showed that fluorescent signal of the blood specimen reached a peak value at the initial administration, and then gradually decreased in either reBiosome^GFP^ group or eBiosome^GFP^ group (Fig. 4e). The difference was that the fluorescent signal of blood specimen in reBiosome^GFP^ group could be detected until 48 h, while that in eBiosome^GFP^ was not detectable after 4 h. The area under the curve (AUC) values for 0 to 48 h of the saline-treated group, eBiosome-treated group and reBiosome-treated group were determined as 35.6 ± 0.6, 42.2 ± 8.0 and 86.4 ± 3.1 × 10^10^ p/cm^2^/sr, respectively. However, these AUC values were interfered by the inherent absorption background in the imaging system. Therefore, a novel parameter ΔAUC by subtracting the AUC of the saline group was introduced to compare the pharmacokinetic characteristics of eBiosome and reBiosome. Accordingly, ΔAUC values of eBiosome group and reBiosome group were 6.6 ± 8.0 and 50.8 ± 3.1 × 10^10^ p/cm^2^/sr, respectively, indicating that reBiosome^GFP^ significantly prolonged the blood circulation time compared with eBiosome^GFP^ (Fig. 4f). The reduced immunogenicity and prolonged blood circulation time of reBiosome can be explained by the mask of PEG polymer on the virus envelope protein inhibits the activation of phagocytes and the immune system and ultimately prolongs the circulation time [43].

### Enhanced Gene Delivery to Weakly Acidic Foci by Virus-Like reBiosome

**Fig. 5:**
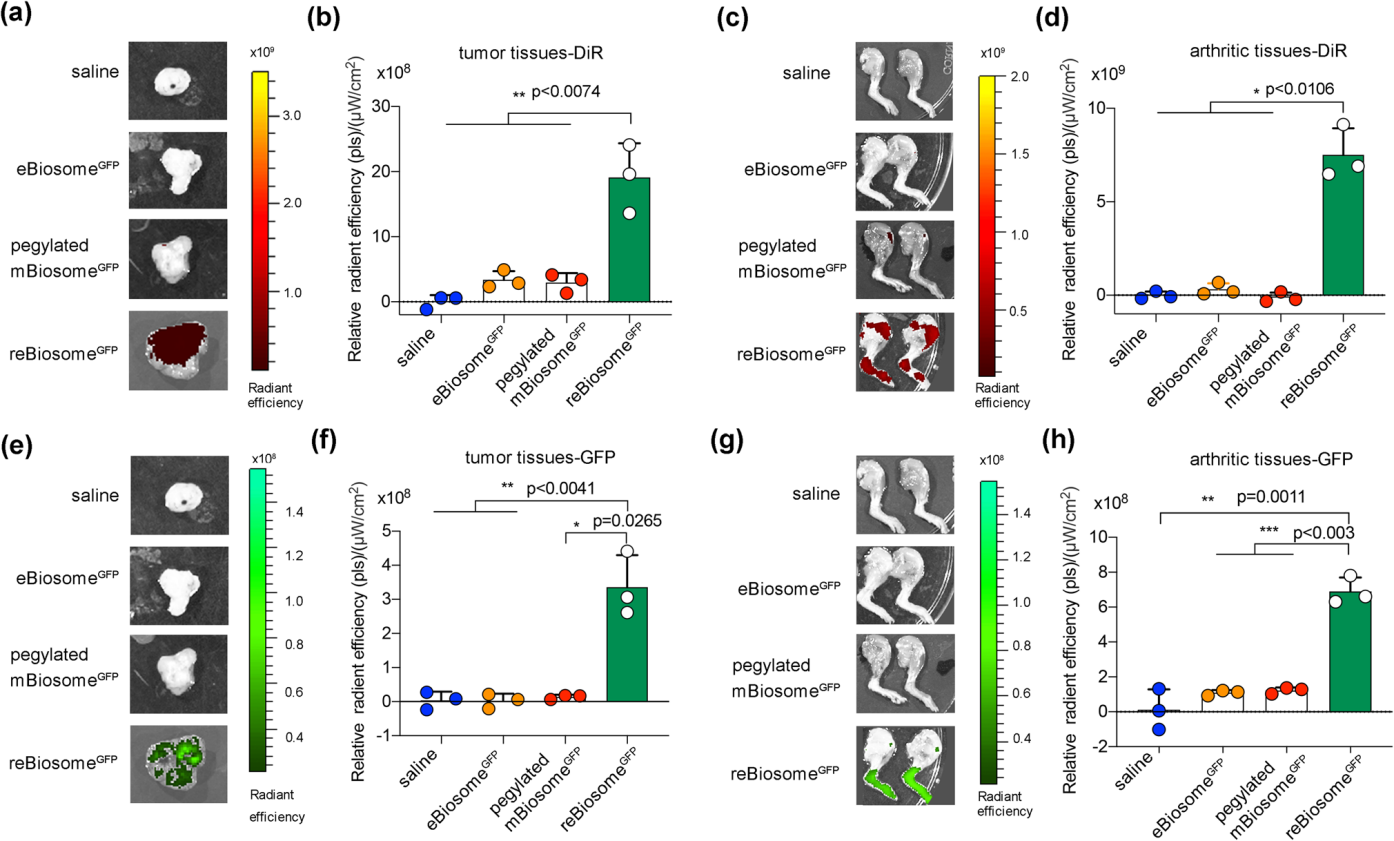
Enhanced gene delivery to weakly acidic foci by virus-like reBiosome. **a**, **b** DiR fluorescence image and the quantification of DiR fluorescence intensity in tumor tissues from breast cancer-bearing mice, respectively. The breast cancer-bearing mice were treated with DiR-labeled reBiosome^GFP^, eBiosomes^GFP^, pegylated mBiosome^GFP^, or saline by intravenous injection. The tumor tissues were collected at 96 h, followed by DiR fluorescence imaging using the LivingImage system. **c**, **d** DiR fluorescence image and the quantification of DiR fluorescence intensity in arthritic tissues from CIA mice, respectively. The CIA mice were treated with DiR-labeled reBiosome^GFP^, eBiosomes^GFP^, pegylated mBiosome^GFP^, or saline. The arthritic tissues were collected at 96 h, followed by DiR fluorescence imaging using the LivingImage system. **e**, **f** Expression of GFP and the quantification of GFP fluorescence intensity in tumor tissues, respectively. **g**, **h** Expression of GFP and the quantification of GFP fluorescence intensity in arthritic tissues, respectively. Data are represented as the mean ± standard deviation (*n* = 3). The two-tailed unpaired Student's t-test was used to determine the significance between groups. (Color figure online)

Evidence has demonstrated that tumor and inflammatory tissues often exhibit a weakly acidic microenvironment [44]. In this study, we established breast cancer-bearing mice and collagen-induced arthritis (CIA) mice models to evaluate the distribution of biosomes and the expression of the GFP gene delivered by biosomes in these weakly acidic foci. The formulations of reBiosome^GFP^, eBiosome^GFP^, and pegylated mBiosome^GFP^ were labeled with DiR. Subsequently, the breast cancer-bearing mice and CIA mice were intravenously treated with these formulations, while saline (PBS pH 7.4) was used as the blank control. After 96 h treatment, the mice were sacrificed, and tumor tissues from breast cancer-bearing mice and arthritic tissues from CIA mice were collected and examined using DiR and GFP fluorescence imaging, respectively. The results obtained from DiR fluorescence imaging demonstrated a robust enhancement in the distribution of reBiosome^GFP^ within tumor tissues compared to eBiosome^GFP^ or pegylated mBiosome^GFP^ (Fig. 5a, b). Similarly, enhanced DiR fluorescence was observed in arthritic tissues among these groups (Fig. 5c, d). Moreover, the results from GFP fluorescence imaging revealed a significantly higher expression level of GFP in weakly acidic tissues (tumor tissues in Fig. 5e, f and arthritic tissues in Fig. 5g, h) from reBiosome^GFP^-treated mice compared to the other groups. These results provide evidence that reBiosome is able to remarkably enhance gene delivery efficiency to weakly acidic foci-like tumor and arthritic tissue. The reason can be explained by two aspects. On one hand, the imidized DBCO-PEG polymer contributes to an increased steric hindrance, enabling the evasion of rapid elimination by the immune system and prolonging the blood circulation time of reBiosomes [43], as verified in Fig. 4. On the other hand, the imidized DBCO-PEG polymer possesses a cleavable nature in the weakly acidic environment, such as tumor or arthritic disease foci. This pH-responsiveness allows reBiosome to selectively release gene cargo in the weakly acidic disease foci.

### Efficacy of reBiosome Packaging Gene Editing System in the Treatment of Breast Cancer

Aside from packaging GFP mRNA, biosomes can be utilized to package therapeutic RNA, and this holds great promise for various applications. To treat breast cancer, a gene editing system composed of Cas9 mRNA and the sgRNA targeting the oncogenic enhancer αE_*myc*_ of c-Myc was encapsulated within different biosomes, resulting in the generation of eBiosome^*α*Emyc^, mBiosome^*α*Emyc^, pegylated mBiosome^*α*Emyc^, and reBiosome^*α*Emyc^ formulations. Our previous study has demonstrated that *α*E_*myc*_ enhanced c-Myc transcription, while deletion of *α*E_*myc*_ led to significant downregulation of c-Myc, effectively inhibiting the proliferation of ER^+^ breast cancer cells [45]. To validate the gene-editing capability of the biosome-packaged Cas9 mRNA and sgRNA targeting *α*E_*myc*_, ER^+^ breast cancer MCF-7 cells were treated with mBiosome^*α*E*myc*^, as reBiosome^αE*myc*^ exhibited limited endocytosis. mBiosome^*α*E*myc*^ could be internalized into MCF-7 cells mediated by LDLR, followed by endosome fusion and the release of the packaged *α*E_*myc*_ gene editing system into the cytoplasm. Subsequently, the system was transported into the nuclei to edit the *α*E_*myc*_ sequence (Fig. S8a). DNA sequencing confirmed the successful deletion of the *α*E_*myc*_ DNA sequence upon mBiosome^*α*E*myc*^ treatment (Fig. S8b). Furthermore, qRT-PCR and Western blot analysis demonstrated a noticeable reduction in c-Myc mRNA transcription and protein expression following the deletion of *α*E_*myc*_ (Fig. S8c, d). The viability assay and apoptosis assay revealed that *α*E_*myc*_ deletion significantly inhibited the proliferation (Fig. S8e) and induced apoptosis (Fig. S8f) in MCF-7 cells. These results confirm the potential of the gene editing system packaged in the biosome to delete the *α*E_*myc*_ sequence, leading to a reduction in c-Myc expression and a significant inhibition of breast cancer cells.

**Fig. 6:**
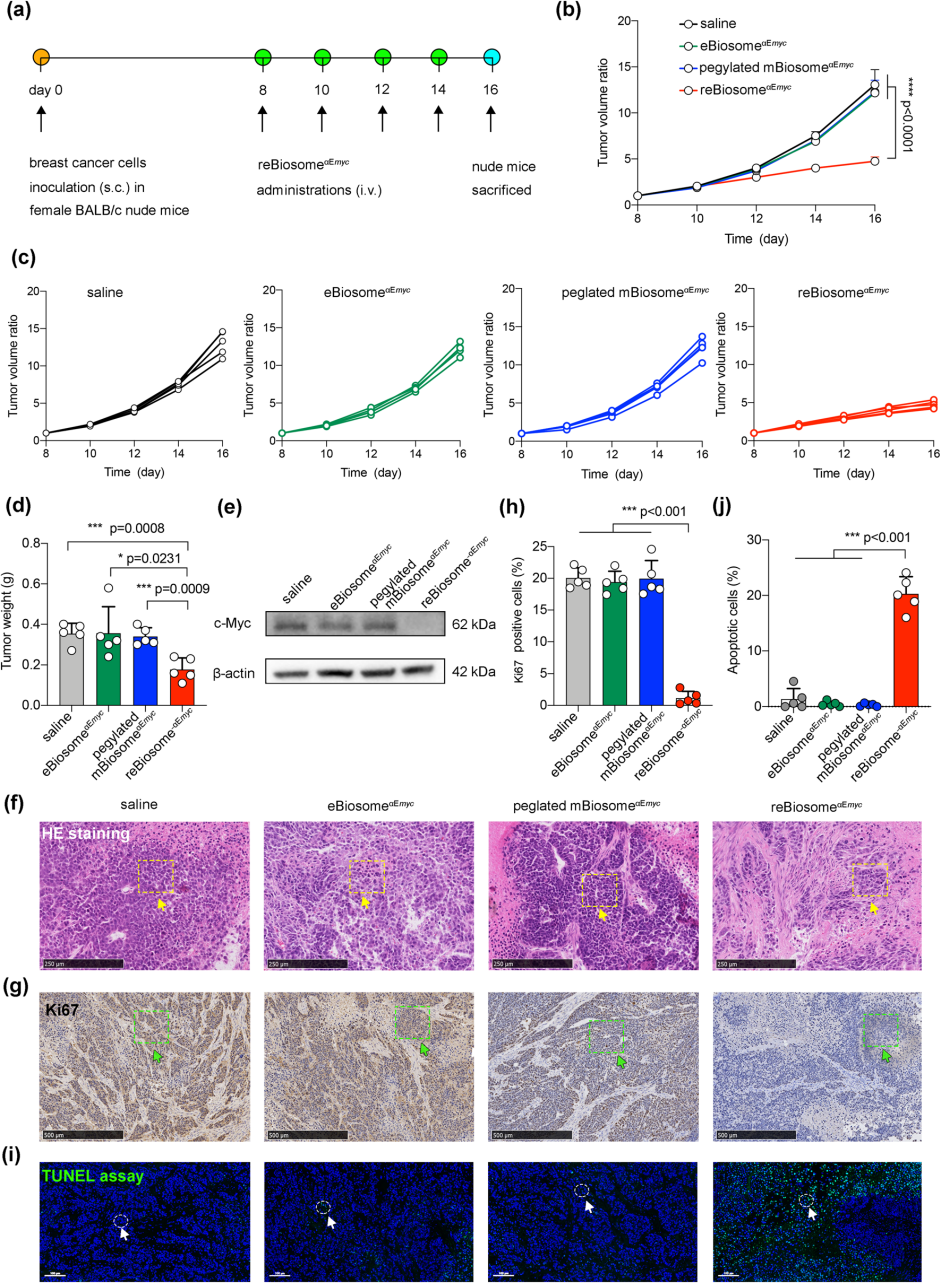
Efficacy of reBiosome packaging gene editing system in the treatment of breast cancer. **a** Schematic representation of the dosing regimen in breast cancer-bearing mice. Saline (PBS pH 7.4), eBiosomes^*α*E*myc*^, pegylated mBiosome^αE*myc*^, and reBiosome^*α*E*myc*^ were intravenously injected into the mice via the tail vein at day 8, 10, 12, and 14, respectively. The mice were sacrificed at day 16. **b** Theraputic efficacy of reBiosome^*α*E*myc*^ in breast cancer-bearing mice. **c** Tumor volume ratios of the formulations. **d** Inhibitory effect of reBiosome^*α*E*myc*^ on tumor weight. **e** Reduced expression of c-Myc protein in tumor tissue by reBiosome^*α*E*myc*^ treatment. The study was performed by Western blot. β-actin was used as the internal reference. **f** HE staining of tumor tissue. Yellow rectangles indicate cancer cells. The scale bar represents 250 μm. **g** Ki67 staining. Green rectangles indicate Ki67 positive cells. The scale bar represents 500 μm. **h** Suppressed proliferation of cancer cells in tumor tissue by reBiosome^*α*E*myc*^ treatment. The percentage of Ki67-positive cells was quantified based on the Ki67 staining images from Fig. 6g. **i** TUNEL assay. Green dots indicate double-strand DNA breaks; blue dots indicate the nucleus; white circles indicate apoptotic cells. The scale bar represents 100 μm. **j** Enhanced apoptosis of cancer cells in tumor tissue by reBiosome^*α*E*myc*^ treatment. The percentage of apoptotic cells was quantified based on the TUNEL images from Fig. 6i. Data are represented as the mean ± standard deviation (*n* = 5). In Fig. 6b, the two-way analysis of variance (ANOVA) was used to determine the significance among groups. In other panels, the two-tailed unpaired Student's t-test was used to determine the significance between groups. (Color figure online)

In order to assess the therapeutic potential of biosome^αE*myc*^ for systemic gene therapy in breast cancer, the breast cancer-bearing mice were treated with saline (PBS pH 7.4), eBiosome^*α*E*myc*^, pegylated mBiosome^*α*E*myc*^, and reBiosome^*α*E*myc*^ via the tail vein, respectively (Fig. 6a). The treatment efficacy of various formulations was evaluated by monitoring the tumor volume. The results revealed that reBiosome^*α*E*myc*^ treatment significantly suppressed tumor growth compared to the controls (Fig. 6b-d). After sacrificing the animals, the tumor tissues were collected and weighed. The results demonstrated that the average tumor weight in the reBiosome^*α*Emyc^-treated group was approximately 50% lighter than that in the control groups (Fig. S9). Furthermore, Western blot analysis confirmed a marked reduction in c-Myc protein expression in tumor tissues following reBiosome^*α*E*myc*^ treatment (Fig. 6e), indicating the successful deletion of the *α*E_*myc*_ enhancer of c-Myc by reBiosome^*α*E*myc*^. Moreover, histological staining and the TUNEL assay provided evidence that reBiosome^αE*myc*^ treatment significantly inhibited proliferation in tumor tissues (Fig. 6f-h) and induced apoptosis of cancer cells within the tumor tissue (Fig. 6i, j). Consequntly, these findings verified the robust efficacy of reBiosome^αE*myc*^ in treating breast cancer-bearing mice, suggesting its potential as an effective gene therapy for breast cancer.

Furthermore, to conduct a preliminary safety assessment of biosome^αE*myc*^, the body weight of each mouse was monitored every other day starting from day 8. After sacrificing the animals at day 16, major organs including the heart, liver, spleen, lung, and at kidney were collected for histochemical analysis. The results revealed that none of the formulations had any noticeable effect on the body weight of the breast cancer-bearing mice (Fig. S10) or on the histological appearance of the major organs (Fig. S11), indicating the absence of significant adverse effects caused by the treatments. This preliminary safety assessment provides an initial evidence for the potential safety of the formulation in the treatment of breast cancer.

### Efficacy of reBiosome Packaging Gene Silencing System in the Treatment of Arthritis

In order to treat rheumatoid arthritis (RA), a gene silencing system utilizing TNFα shRNA was encapsulated within different biosomes, resulting in the generation of eBiosome^TNF*α*^, mBiosome^TNF*α*^, pegylated mBiosome^TNF*α*^ and reBiosome^TNF*α*^. It is known that TNFα is primarily secreted by macrophages, and plays a critical role in the pathophysiological processes associated with RA [46]. By suppressing TNFα production in macrophages, it is possible to induce a polarization from a pro-inflammatory (M1) state to an anti-inflammatory (M2) state, thereby alleviating RA symptoms. To validate the gene-silencing capability of the biosome-packaged TNF*α* shRNA, lipopolysaccharide (LPS)-treated murine macrophages (RAW 264.7 cells) were treated with mBiosome^TNF*α*^, as reBiosome^TNF*α*^ showed limited endocytosis. mBiosome^TNF*α*^ could be internalized into LPS-treated macrophages mediated by LDLR, followed by endosome fusion and the release of the packaged TNF*α* gene silencing system into the cytoplasm, resulting in the degradation of TNF*α* mRNA (Fig. S12a). ELISA analysis revealed that mBiosome^TNF*α*^ treatment significantly reduced the concentration of TNFα in the culture supernatant of macrophages (Fig. S12b). Moreover, flow cytometry measurements of CD206 (a marker protein for M2-type macrophages) demonstrated that mBiosome^TNFα^ treatment led to a significant increase in CD206 expression compared to the control groups, indicating a polarization toward anti-inflammatory macrophages (M2-type, Fig. S12c). These findings suggest the potential of the TNF*α* gene silencing system packaged in the biosome to inhibit TNF*α* secretion by macrophages and promote macrophage polarization toward an M2 anti-inflammatory state.

**Fig. 7:**
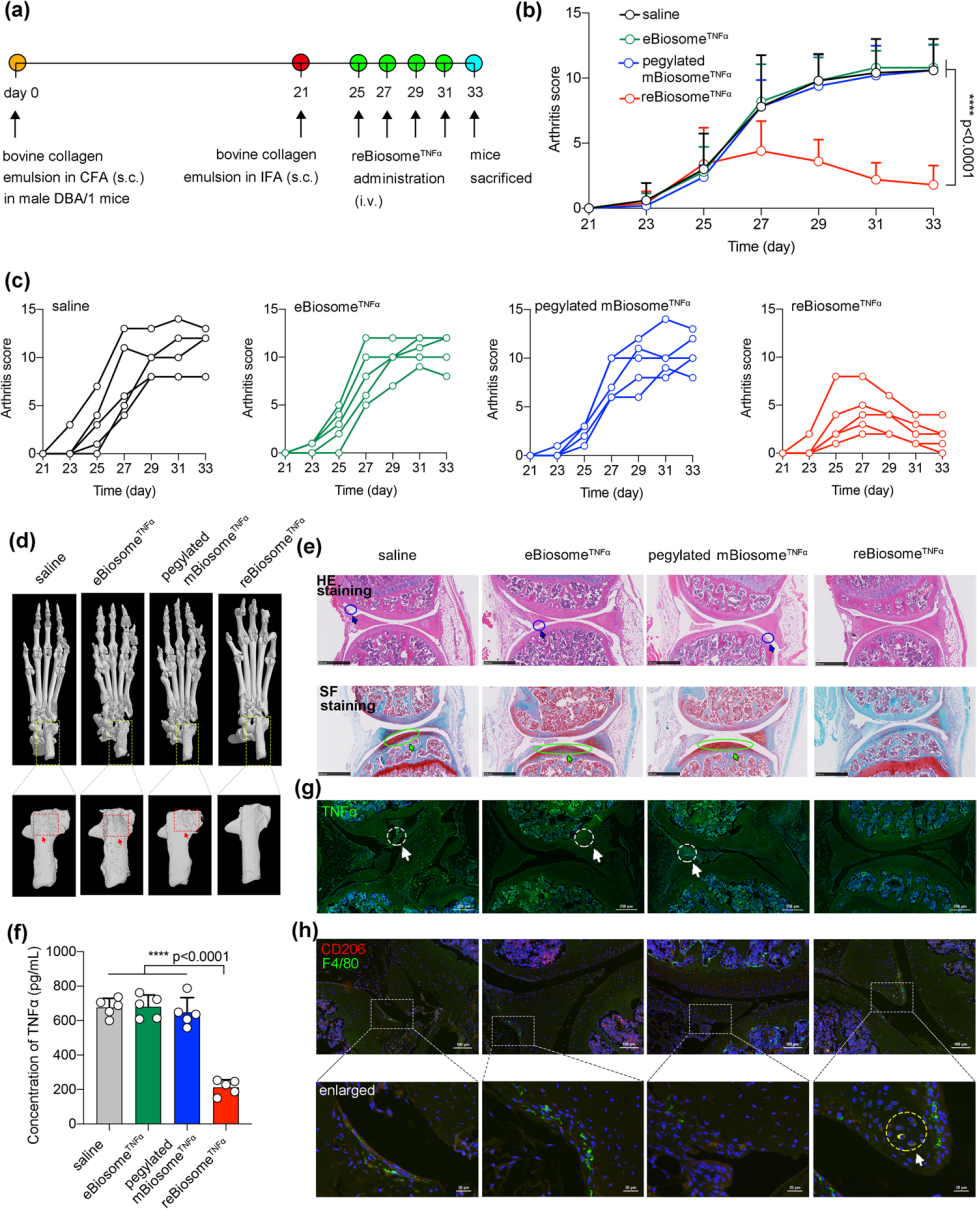
Efficacy of reBiosome packaging gene silencing system in the treatment of arthritis. **a** The illustration for the dosing scheme of reBiosome^TNF*α*^ in arthritic mice. At day 25, day 27, day 29, and day 31, saline (PBS pH 7.4), eBiosomes^TNF*α*^, pegylated mBiosome^TNF*α*^, and reBiosome^TNF*α*^ were intravenously injected into the mice via the tail vein, respectively. At day 33, mice were sacrificed. **b** Therapeutic efficacy of reBiosome^TNFα^ in arthritic mice based on arthritis scores. The arthritis scores of the four groups were recorded every other day. **c** Arthritis scores of varying formulations. **d** Micro-CT images of arthritic mouse paws. The left hind paws (top) and calcaneus (bottom) from each animal after sacrifice were scanned by micro-CT (computer tomography). Red rectangles indicate osteolysis. **e** Immunostaining of knee joints. **Top**: HE staining. Scale bar, 500 μm. Blue circles indicate synovial membrane fibrillation. **Bottom**: SF staining. Scale bar, 500 μm. Green circles indicate cartilage hyperplasia. **f** Reduced TNFα levels in blood circulation by reBiosome^TNF*α*^ treatment. At day 33, blood specimens were collected from each animal to measure the concentration of TNF*α* by ELISA. **g** Decreased TNF*α* levels in knee joints by reBiosome^TNF*α*^ treatment. Green, TNFα; blue, nucleus. Scale bar, 250 μm. White circles indicate high expression of TNFα. **h** Polarization of macrophages in knee joints by reBiosome^TNF*α*^ treatment. Green, F4/80; red, CD206. The yellow circle indicates macrophages in M2-type. Scale bar, 100 μm (top) or 20 μm (bottom). Data are represented as the mean ± standard deviation (*n* = 5). In Fig. 7b, the two-way analysis of variance (ANOVA) was used to determine the significance among groups. In Fig. 7f, the two-tailed unpaired Student's t-test was used to determine the significance between groups. (Color figure online)

To evaluate the systemic gene therapy efficacy of biosome^TNF*α*^ in rheumatoid arthritis (RA) in vivo, we employed collagen-induced arthritis (CIA) mouse model, a well-established model for studying RA [47]. In this model, arthritis symptoms are often observed at day 21–28 since the initial immunization [48]. Accordingly, the joint inflammation signs were monitored every other day starting from day 21. At day 25, CIA mice were treated with saline (PBS pH 7.4), eBiosome^TNF*α*^, pegylated mBiosome^TNF*α*^, and reBiosome^TNF*α*^ via the tail vein, respectively (Fig. 7a). The efficacy of different formulations was assessed by comparing the joint inflammation signs after treatment. The results revealed that reBiosome^TNF*α*^ treatment significantly alleviated erythema and swelling in the joints (Fig. S13a, b) and reduced arthritis scores compared to other groups (Fig. 7b, c). Moreover, we conducted micro-CT and histological staining studies to evaluate the anti-inflammatory effect in the ankle and knee joints of arthritic mice, respectively. The results demonstrated that reBiosome^TNFα^ treatment relieved osteolysis (Fig. 7d), synovial membrane fibrillation, and cartilage hyperplasia (Fig. 7e). Furthermore, the content of TNF*α* in the blood and knee joints were tested using ELISA and immunofluorescence, respectively. The results showed that reBiosome^TNFα^ treatment significantly reduced TNFα content both in the blood (Fig. 7f) and knee joints (Fig. 7g). In addition, immunofluorescence analysis revealed that reBiosome^TNFα^ treatment markedly enhanced CD206 expression in macrophages compared to other groups, indicating polarization toward M2-type macrophages (Fig. 7h). These findings demonstrate that reBiosome^TNF*α*^ exhibits robust efficacy in treating CIA mice, revealing its potential as an effective treatment for rheumatoid arthritis.

To conduct a preliminary safety evaluation of biosomes^TNF*α*^, we monitored the body weight of each mouse every other day starting from day 21, and upon sacrificing the animals at day 33, major organs were collected for histochemical analysis. The results indicated that none of the formulations had any significant impact on the body weight of the arthritic mice (Fig. S14). Moreover, histological staining of the major organs including the heart, liver, spleen, lung, and kidney revealed no observable abnormalities or adverse effects caused by the treatments (Fig. S15). These findings suggest that the administration of the tested formulations did not result in any apparent alterations in body weight or induce histopathological changes in the major organs of the arthritic mice. This preliminary safety evaluation provides an initial evidence for the potential safety of this formulation for the treatment of rheumatoid arthritis.

## Conclusions

In this study, we successfully engineered a virus-like nanoparticle named reBiosome by site-specific codon mutation for displaying Azi on VSV-G of eBiosome at a rational site, followed by incorporating weak acid-responsive PEG polymer via bioorthogonal chemistry. The results showed that the reBiosome demonstrated reduced immunogenicity, prolonged blood circulation time and realized enhanced gene delivery to weakly acidic foci (like tumor and arthritic tissues) in mice. Moreover, reBiosome demonstrated remarkable therapeutic efficacy in breast cancer after packaging the gene editing system, and in arthritis after packaging the gene silencing system, respectively. As a versatile gene delivery platform, reBiosome holds the potential for broad applications, such as the use for the treatment of various solid cancers like pancreas cancer and colon cancer, and for the treatment of various inflammatory diseases like atherosclerosis and asthma. In conclusion, this study developed a novel bioorthogonal engineered virus-like reBiosome, which could offer a promising and universal gene therapy platform for the treatment of major diseases such as cancer and inflammatory disorders.

### Supplementary Information

Below is the link to the electronic supplementary material.

Supplementary file1 (DOCX 24203 KB)

Supplementary file2 (ZIP 256 KB)

Supplementary file3 (XLSX 16 KB)
