# Supplementary material for: Bioorthogonal Engineered Virus-Like Nanoparticles for Efficient Gene Therapy
Source: Nanomicro Lett. 2023 Aug 12;15:197. doi: 10.1007/s40820-023-01153-y (PMC10423197; doi:10.1007/s40820-023-01153-y)
Supplement: Supplementary file 1 — Supplementary file1 (DOCX 24203 KB) [file 40820_2023_1153_MOESM1_ESM.docx]

Supplementary Information for

Bioorthogonal engineered virus-like nanoparticles for efficient gene therapy

Chun-Jie Bao^1,2,3†^, Jia-Lun Duan^1,2†^, Ying Xie^1†^, Xin-Ping Feng^4^, Wei Cui^4^, Song-Yue Chen^1^, Pei-Shan Li^1^, Yi-Xuan Liu^1^, Jin-ling Wang^1^, Gui-Ling Wang^1^, Wan-Liang Lu^1*^

This PDF file includes:

Supplementary Materials and Methods

Supplementary Results

Figures S1 to S18

Tables S1 to S2

Legend for Dataset S1

Legend for Dataset S2

SI References

Other supplementary materials for this manuscript include the following:

Dataset S1 to Dataset S2

**Supplementary Materials and Methods**

**Cells**

Human embryonic kidney 293T (HEK 293T) cells, human breast cancer MCF-7 cells, and murine macrophage RAW 264.7 cells were purchased from the Institute of Basic Medical Sciences, Chinese Academy of Medical Sciences (Beijing, China). HEK 293T cells and RAW 264.7 cells were cultured in Dulbecco’s modified Eagle’s medium (Macgene, Beijing, China) at 37°C under 5% CO_2_. MCF-7 cells were cultured in Roswell Park Memorial Institute (RPMI) 1640 medium (Macgene, Beijing, China) at 37°C under 5% CO_2_. These culture media were supplemented with 10% fetal bovine serum (FBS; PAN, Beijing local agent, Germany), 100 U/mL penicillin (Macgene, Beijing, China), 100 μg/mL streptomycin (Macgene, Beijing, China) and 1× mycoplasma elimination reagent (#40607, Yeason, Shanghai, China).

**Plasmid construction**

pCMV-VSV-G (plasmid #8454), gag-poI (plasmid #14887), and pIRE4-Azi (plasmid #105829) were purchased from Addgene (Beijing local agent, China). pCMV-VSV-G-D192, pCMV-VSV-G-A246, pCMV-VSV-G-A247, pCMV-capsid10-flag, pCMV-cargoGFP, pCMV-cargoCas9, pCMV-cargoshTNFα, pCMV-cargoshcontrol, and pLKO-sgRNA were constructed by Tsingke Biotechnology (Beijing, China) (Table S1 and Dataset S1).

**Materials**

Polyethylene glycol methyl ether aldehyde (mPEG_2000_-CHO), dibenzocyclooctyne-polyethylene glycol (DBCO-PEG_2000_), and CDCl_3_ were purchased from Shanghai Aladdin Bio-Chem Technology (Shanghai, China). Dibenzocyclooctyne-amine (DBCO-NH_2_) was purchased from Tanshtech (Guangzhou, China). Superdry dichloromethane (CH_2_Cl_2_), pyrrolidine, and a 4 Å molecular sieve were purchased from Energy Chemical (Shanghai, China). Sephadex LH-20 was purchased from Solarbio Life Sciences (Beijing, China). A C18 column (5 μm, 4.6 x 250 mm) was purchased from Agilent Technologies (Beijing local agent, China). PBS was purchased from Macgene (Beijing, China). Methanol (HPLC grade) and other chemical reagents (reagent grade) were purchased from Beijing Tongguang Fine Chemicals Company (Beijing, China). Milli-Q water was obtained from a Millipak filter unit (Millipore, pore size 0.22 μm). DiI (1,1'-dioctadecyl-3,3,3',3'-tetramethylindocarbocyanineperchlorate) and DiR (1,1-dioctadecyl-3,3,3,3-tetramethylindotricarbocyanine iodide) was purchased from Yeason Biotech (Shanghai, China). A TNFα ELISA kit (#P06804) was purchased from Cusabio (Wuhan, China). Lipopolysaccharide (LPS) was purchased from Bioss (Beijing, China). Type II bovine collagen, complete Freund’s adjuvant (CFA), and incomplete Freund’s adjuvant (IFA) were purchased from Chondrex (Beijing local agent, China).

**Antibodies**

Anti-VSV-G antibody (#V4888), and anti-LDL receptor antibody (#ZRB1176) were purchased from Sigma-Aldrich (Beijing local agent, China). Anti-c-Myc antibody (#5605) and anti-β-actin antibody (#4970) were purchased from Cell Signaling Technology (Beijing local agent, China). FITC anti-mouse F4/80 antibody (#E-AB-F0995C) and APC anti-mouse CD206 antibody (#E-AB-F1135E) for flow cytometry were purchased from Elabscience Biotechnology Co., Ltd. (Beijing local agent, China). Alexa Fluor 488-conjugated secondary antibody (#A-11059) and Alexa Fluor 647-conjugated secondary antibody (#A-21235) were purchased from Invitrogen (Beijing local agent, China). Anti-Ki67 primary antibody (#GB121141), anti-F4/80 primary antibody (#GB113373), anti-CD206 primary antibody (#GB113497), anti-TNFα primary antibody (#GB11188), HRP-conjugated secondary antibody (#GB23301), FITC-conjugated secondary antibody (#GB22303), and Cy5-conjugated secondary antibody (#GB27303) for immunohistochemistry and immunofluorescence were purchased from Servicebio (Wuhan, China).

**Preparation of mutated VSV-G protein**

For the preparation of mutated VSV-G proteins (mProteins), HEK 293T cells were cultured in 25 cm^2^ flasks at 80-90% confluence and were transfected with 2 μg pIRE4-Azi, 4 μg pCMV-VSV-G or a mutated VSV-G expression plasmid (pCMV-VSV-G-D192, pCMV-VSV-G-A246 or pCMV-VSV-G-A247) by Polyjet (Unique Biotechnology Company, Beijing, China) according to the manufacturer’s instructions. The medium was supplemented with 1 mM 4-azido-L-phenylalanine (Azi) purchased from MedChemExpress (Monmouth Junction, NJ, USA).

**LC-MS/MS peptide sequencing for mProteins**

For the characterization of the display of Azi at the site-specific site, mProteins were purified by immunoprecipitation (IP) and analyzed by liquid chromatography with tandem mass spectrometry (LC-MS/MS). Briefly, total protein was extracted and incubated with anti-VSV-G antibody and protein A/G agarose (Invitrogen, Beijing local agent) to obtain the IP product according to the manufacturer’s instructions. The IP product was separated by 10% sodium dodecyl sulfate-polyacrylamide gel electrophoresis (SDS-PAGE) and visualized with Coomassie blue staining. The corresponding protein bands were sliced and digested in gel with sequencing-grade trypsin at 37°C overnight. The digested peptides were extracted twice with 60% acetonitrile-water solution (containing 5% trifluoroacetic acid, v/v) and once with pure acetonitrile and then concentrated by vacuum. The concentration of protein was determined by microspectrophotometry (Nano300, YPG-Bio, Beijing, China). The digested peptides were separated by gradient elution at a flow rate of 0.300 µL/min for 120 min by LC-MS/MS (Ultimate 3000 nano-UPLC-Fusion LUMOS mass spectrometer, Thermo Fisher, Waltham, USA). The analysis was performed on an Acclaim PepMap RSLC column (75 µm ID, 250 mm length, C18). Mobile phase A consisted of 99.9% water and 0.1% formic acid (v/v), and mobile phase B consisted of 99.9% acetonitrile and 0.1% formic acid (v/v). The Fusion LUMOS mass spectrometer was equipped with a data-dependent acquisition mode using Xcalibur 4.1.50 software and was fully scanned by Orbitrap (375-1500 m/z, 60,000 resolution). MS/MS spectra from each run were searched against the selected database using the software Proteome Discovery (version 2.2).

**Molecular dynamics simulation**

The three-dimensional structure of vesicular stomatitis virus glycoprotein G (VSV-G) in complex with the CR2 domain of the low-density lipoprotein receptor (LDLR) was taken from the RCSB protein data bank (PDB ID code 5OYL), and then, three mutations (D192, A246, and A247) were introduced to the protein sequence using the Tleap module of the AMBER 16 program.

Partial atomic charges of modified amino acids were derived using the restrained electrostatic potential (RESP) charge scheme, and amber atom types were matched with the GAFF [1] database by the antechamber program of AmberTools 16 software. [2] The complex was dissolved in a rectangular box filled with the TIP3P explicit water model [3] fitted to protein size, with a minimum distance of 12.0 Å between the protein surface and the box edges. The required amount of Na^+^ counter ions were added to balance the charge deficit of the system.

For each independent replica of molecular dynamics, the SHAKE algorithm was used to restrain the mobility of hydrogen atoms. The molecular dynamics simulation was conducted by PMEMD.CUDA (Particle Mesh Ewald Molecular Dynamics) module integrated into AMBER 16. The whole system was first minimized and then heated from 0 K to 310 K for 60 ps, followed by stabilization at 310 K for 20 ps and the production of MD data for 50 ns.

The time evolutions of root mean square deviation (RMSD) and the secondary structure were analyzed by the CPPTRAJ module of AMBER 16 based on MD trajectory. MM/GBSA technique implemented in AMBER 16 was used to calculate the free energy between D192 and its surrounding residues, where ΔEele (electrostatic) term was calculated by the sander module of AMBER 16. For each complex, 500 snapshots were taken from 45 to 50 ns on the MD trajectory. Further, the total free energy for varying systems was decomposed into D192-residue pairs using the MM/GBSA decomposition analysis by the mm pbsa program [4] in AMBER 16. The electrostatic term energy contribution was taken into consideration.

The topography and electrostatic potential distribution of receptor molecular surface were characterized using the molecular modeling package Sybyl-X 2.0. The visualization of spatial surface profiles was performed through Connolly program. [5, 6] MOLCAD program was also used to generate Gasteiger electrostatic potential maps on the molecular surface.

**Preparation of varying biosome^GFP^**

For the preparation of varying biosome packaging the GFP expressing system (eBiosome^GFP^, mBiosome^GFP^ D192, mBiosome^GFP^ A246, and mBiosome^GFP^ A247), pCMV-cargoGFP was constructed by cloning cargoGFP (5'UTR-GFP-3'UTR, GFP sequence grafted with 5' UTR and 3' UTR of capsid 10) into the pCMV plasmid backbone (Dataset S1). HEK 293T cells were cultured in 225 cm^2^ flasks at 80-90% confluence, followed by transfection with 15 μg VSV-G coding plasmid (pCMV-VSV-G, pCMV-VSV-G-D192, pCMV-VSV-G-A246, or pCMV-VSV-G-A247), 7.5 μg pIRE4-Azi, 15 μg pCMV-capsid10-flag, and 15 μg pCMV-cargoGFP by Polyjet according to the manufacturer’s instructions. The medium was supplemented with 1 mM Azi. The cell supernatant was collected twice at 48 h and at 72 h post-transfection. The supernatant was purified by centrifugation at 10000 × g for 1 h to remove cell debris and macro vesicles, followed by ultracentrifugation at 50000 g at 4°C for 2 h using an ultracentrifuge (Beckman Coulter, FL, USA). The supernatant was decanted, and the remaining biosome^GFP^ pellets were resuspended in PBS (pH 7.4) for further analysis.

**Quantification of the gene cargoes packaged in biosome**

For the establishment of a universal quantification method for the gene cargoes packaged in biosome, a fragment from the 5' UTR of capsid 10 (5' frag) was first amplified from the plasmid pCMV-capsid10-flag by polymerase chain reaction (PCR). To establish the standard curve between the Cq and the concentration of 5' frag, a series of concentrations of 5' frag DNA standard was analyzed with quantification primers (Table S4, Supporting Information) by quantitative real-time PCR (qRT-PCR) using the reagent mix (#LV900-2, Applied Biological Materials Inc., Richmond, BC, Canada). For quantifying the 5' frag content, biosomes were lysed by virus lysis buffer (#LV900-1, Applied Biological Materials Inc., Richmond, BC, Canada), and the gene cargoes in the lysate were reversed and analyzed with the above quantification primers using the reagent mix. The content of gene cargoes was indicated by the concentration of the 5' frag packaged in biosome. A representative quantification template is given in Dataset S2.

**Synthesis and separation of imidized DBCO-PEG**

Imidized DBCO-PEG was synthesized via a Mannich dehydration reaction. Briefly, 40 mg DBCO-NH_2_ (1 equiv) and 288 mg mPEG_2000_-CHO (1 equiv) were pre-dissolved in super-dried CH_2_Cl_2_ and added into a 15 mL heavy-wall pressure vessel. Then, pyrrolidine (0.1 equiv) and a 4 Å molecular sieve were added to the vessel. The vessel was sealed and heated at 40°C for 2 h. For the separation of the imidized DBCO-PEG, the reaction mixture was filtered, evaporated, and resuspended in methanol. Then, the imidized DBCO-PEG was fractionated by Sephadex LH-20 column chromatography and further evaporated to powders.

**Characterization of imidized DBCO-PEG**

For the characterization of the molecular weight distribution of imidized DBCO-PEG, 1 mg imidized DBCO-PEG, and 1 mg mPEG_2000_-CHO were dissolved in 100 μL of methanol and measured by MALDI-TOF mass spectrometry (AB SCIEX, Singapore). The molecular weight range of imidized DBCO-PEG was estimated by the mass/charge (m/z) ratios.

For the characterization of the structure of imidized DBCO-PEG, 50 mg imidized DBCO-PEG, 50 mg mPEG_2000_-CHO and 50 mg DBCO-NH_2_ were dissolved in 500 μL of CDCl_3_ and verified by nuclear magnetic resonance (NMR, Bruker, 600 Hz) via ^1^H NMR and ^13^C NMR. NMR spectra were analyzed by iNMR 6 (Nucleomatica). Imidized DBCO-PEG, mPEG_2000_-CHO, and DBCO-NH_2_ were diluted with absolute ethyl alcohol, and the solutions were loaded onto KBr crystals. The infrared absorption data were measured by Fourier transform infrared spectrometry (FT-IR, Thermo Nicolet Nexus 470, Nicolet, USA). Spectra are presented as percent absorbance versus the frequency of absorption (cm^-1^).

**Weakly acid-responsiveness of imidized DBCO-PEG**

For the measurement of the hydrolytic efficiency of imidized DBCO-PEG under weakly acidic medium treatment, imidized DBCO-PEG was dissolved in PBS (pH 7.4, pH 6.5, and pH 5.0) at a final concentration of 7.2 mg/mL, followed by incubation in a water bath at 37°C. At a fixed time-point (0 h, 1 h, 2 h, 4 h, 8 h, 12 h, 24 h, 36 h, and 48 h), the hydrolytic process was stopped by diluting the solution with PBS (pH 7.4). The diluted solution was measured by high-performance liquid chromatography (HPLC, Agilent, 1260 series, CA, USA) to detect the content of imidized DBCO-PEG. The analysis was performed on a C18 column with an ultraviolet (UV) detector at 25°C. The mobile phase consisted of 50% methanol and 50% water (v/v). The detection wavelength was set at 210 nm, and the flow rate was 0.5 mL/min. The content of DBCO-NH_2_ at 0 h (C_0_) or at a fixed time point (C_t_) was detected. The hydrolytic efficiency (h) at t h was calculated by the following equation:

h % = (1-C_t_/C_0_) ×100%

**Preparation of reBiosome^GFP^**

For the preparation of the reBiosome packaging GFP expressing system (reBiosome^GFP^), the above mBiosome^GFP^ A247 was mixed with imidized DBCO-PEG solution at 37°C for 2 h. The resultant reBiosome^GFP^ was further purified by ultracentrifugation at 50000 g at 4°C for 2 h. The supernatant was decanted, and the remaining reBiosome^GFP^ pellets were resuspended in PBS (pH 7.4) for further analysis.

**Hydrolysis of reBiosome^GFP^**

The above reBiosome^GFP^ pellets were resuspended in PBS (pH 6.5) and incubated at 37°C for 12 h. The weak acid-treated reBiosome^GFP^ suspensions were purified by ultracentrifugation at 50000 g at 4°C for 2 h again for further analysis.

**Dynamic light scattering analysis**

For the characterization of the sizes and zeta potential values, varying biosome^GFP^ (eBiosome^GFP^, mBiosome^GFP^ D192, mBiosome^GFP^ A246, mBiosome^GFP^ A247, reBiosome^GFP^, and hydrolyzed reBiosome^GFP^) were measured using dynamic light scattering (DLS) with a Nano Series Zenith 4003 Zetasizer (Malvern Instruments Ltd., Malvern, UK).

**Transmission electron microscopy**

For characterization of the morphology of varying biosome^GFP^ (eBiosome^GFP^, mBiosome^GFP^ D192, mBiosome^GFP^ A246, mBiosome^GFP^ A247, reBiosome^GFP^, and hydrolyzed reBiosome^GFP^), 5 μL of biosome^GFP^ was loaded onto a carbon-coated copper grid and stained with uranyl acetate solution (1% w/v) for 1 min. After drying overnight, the copper grid was observed by transmission electron microscopy (TEM) at 100 kV (JEM-1400, JEOL, Tokyo, Japan).

**Surface plasmon resonance**

For determination of the specific binding of varying biosome^GFP^ (eBiosome^GFP^, mBiosome^GFP^ D192, mBiosome^GFP^ A246, mBiosome^GFP^ A247, reBiosome^GFP^, and hydrolyzed reBiosome^GFP^) with the receptor LDLR, the study was performed by surface plasmon resonance (SPR BIAcore T200, GE Healthcare, MA, USA) with a CM5 sensor chip (GE Healthcare). Briefly, 3000 U of LDLR (Sino Biological Inc., Beijing, China) in acetate buffer (pH 5.0) was immobilized on the chip. Then, biosome suspensions (all packaged with 1 ng 5' frag) were injected into the SPR system at a flow rate of 30 μL/min. The running buffer was PBS (pH 7.4). The association time was 120 s, and the dissociation time was 200 s. Afterward, the chip was regenerated for 30 s with NaOH (10 mM). The chip was equilibrated with the running buffer for 10 s before the next injection.

**Co-localization of varying biosome^GFP^ with LDLR**

MCF-7 cells (4 ×10^4^) were seeded in confocal dishes and incubated under 5% CO_2_ at 37°C overnight, and the cells were transfected with varying biosome^GFP^ (eBiosome^GFP^, mBiosome^GFP^ D192, mBiosome^GFP^ A246, mBiosome^GFP^ A247, reBiosome^GFP^ and hydrolyzed reBiosome^GFP^, all packaging with 1 ng 5' frag) for 2 h, followed by fixation with 4% paraformaldehyde for 15 min and permeabilization with 0.1% Triton X-100 in PBS (pH 7.4) for 15 min. Then, the cells were incubated with the anti-VSV-G antibody (1:500) at 4°C overnight and incubated with the secondary antibody conjugated with Alexa 647 (1:600) at 4°C for 2 h. Afterward, the cells were incubated with the anti-LDLR antibody (1:500) at 25°C for 1 h and then incubated with the secondary antibody conjugated with Alexa 488 (1:600) at 4°C for 2 h. Finally, the cells were stained with Hoechst 33342 (5 μg/mL) at 25°C for 15 min and measured by ultrahigh-resolution confocal laser scanning microscopy (Zeiss LSM880, Oberkochen, Germany) with a 63×oil immersion lens. Specific laser channels consisted of a 405 nm diode, 488 nm argon, and 647 nm diode laser. The laser intensity and detector gain settings were maintained across all image acquisitions for each experiment. The Pearson’s correlation coefficient was calculated by ZEN 2.3 lite software.

**Uptake of varying biosome^GFP^**

For the analysis of uptake, varying biosome^GFP^ (eBiosome^GFP^, mBiosome^GFP^ D192, mBiosome^GFP^ A246, mBiosome^GFP^ A247, reBiosome^GFP^ and hydrolyzed reBiosome^GFP^, all packaging with 10 ng 5' frag) were labeled with DiI by incubation at 25°C for 5 min. HEK 293T cells (2 × 10^5^) were seeded in 6-well plates and cultured under 5% CO_2_ at 37°C overnight. Afterward, the cells were transfected with DiI-labeled biosome^GFP^ for 4 h, 8 h, 12 h or 24 h. After transfection, the cells were collected at a fixed time point, rinsed twice with PBS (pH 7.4), and analyzed at 488 nm by FACScan flow cytometry (Becton Dickinson, San Jose, USA). The uptake rates of the above biosome^GFP^ at varying time points were calculated by FlowJo software.

**Transfection efficiency of varying biosome^GFP^**

HEK 293T cells (2 × 10^5^) were seeded in 6-well plates, cultured under 5% CO_2_ at 37°C overnight, and transfected with varying biosome^GFP^ (eBiosome^GFP^, mBiosome^GFP^ D192, mBiosome^GFP^ A246, mBiosome^GFP^ A247, reBiosome^GFP^ and hydrolyzed reBiosome^GFP^, all packaging with 10 ng 5' frag) for 12 h. Afterward, the culture medium was replaced with DMEM complete medium, and the cells were further incubated for 36 h. Finally, the cells were collected, rinsed twice with PBS (pH 7.4), and analyzed at 488 nm by FACScan flow cytometry.

**Western blot**

For the analysis of the expression of a target protein, total protein was measured by Western blot assays. The target proteins consisted of the mutated VSV-G protein expressed on HEK 293T cells and biosome, the c-Myc protein expressed on MCF-7 cells, and the c-Myc protein expressed on cancer cells isolated from breast cancer-bearing mice. For analyzing cellular proteins, the specified cells were collected and lysed in RIPA buffer (Applygen, Beijing, China) with protease phosphatase inhibitor (Beyotime, Shanghai, China). The cell lysate was centrifuged at 12000 rpm at 4°C for 20 min, and the total protein was collected from the supernatant. For analyzing membrane proteins incorporated in biosomes, the concentrate of biosomes was lysed by membrane protein lysis buffer A (Beyotime, Shanghai, China). Then, the membrane was collected by centrifugation at 12000 rpm at 4°C for 30 min, and treated with membrane protein lysis buffer B (Beyotime, Shanghai, China) on ice for 10 min. Afterward, the lysate was centrifuged at 12000 rpm at 4°C for 5 min, and the membrane protein was collected from the supernatant. Then, the total protein was quantified using a bicinchoninic acid (BCA) protein assay kit (Beyotime, Shanghai, China) according to the manufacturer's instructions and further separated by sodium dodecyl sulfate-polyacrylamide gel electrophoresis (SDS–PAGE). The separated proteins were transferred onto polyvinylidene fluoride (PVDF) membranes (Merck-Millipore, Darmstadt, Germany). The blots on PVDF membranes were blocked with 5% bovine serum albumin (BSA) in TBS-T (Tris-buffered saline with Tween 20) solution, incubated with the appropriate primary antibodies at 4°C overnight, and further incubated with horseradish peroxidase (HRP)-conjugated secondary antibodies at 25°C for 1 h. Finally, the blots were observed by a MiniChemi610 imaging system (Sage Creation, Beijing, China).

**Preparation of varying biosome^αEmyc^**

pCMV-cargoCas9 was constructed by cloning cargoCas9 (5'UTR-Cas9-3'UTR, Cas9 sequence grafted with the 5' UTR and 3' UTR of capsid 10) into the pCMV plasmid. pLKO-sgRNA was constructed by cloning the sgRNA for deleting αE_myc_ into the pLKO plasmid backbone. The sequences of pCMV-cargoCas9 plasmid and pLKO-sgRNA plasmid were given in Dataset S1, and the sequence of sgRNA for αE_myc_ was the same as the sequence of sgRNA 1 in reference [7] and given in Table S2.

For the preparation of biosome packaging the αE_myc_ gene editing system (eBiosome^αEmyc^ and mBiosome^αEmyc^), HEK 293T cells were cultured in 225 cm^2^ flasks at 80-90% confluence, followed by transfection with 15 μg VSV-G encoding plasmid (pCMV-VSV-G or pCMV-VSV-G-A247), 7.5 μg pIRE4-Azi, 15 μg pCMV-capsid10-flag, 7.5 μg pCMV-cargoCas9, and 7.5 μg pLKO-sgRNA by Polyjet according to the manufacturer’s instructions. The medium was supplemented with 1 mM Azi. The cell supernatant was collected twice at 48 h and at 72 h post-transfection. The supernatant was purified by centrifugation at 10000 × g for 1 h to remove cell debris and macro vesicles, followed by ultracentrifugation at 50000 g at 4°C for 2 h. The supernatant was decanted, and the remaining mBiosome^αEmyc^ pellets were resuspended in PBS (pH 7.4) for further analysis.

For the preparation of the mBiosome packaging nonspecific gene editing system (mBiosome^Cas9^), HEK 293T cells were cultured in 225 cm^2^ flasks at 80-90% confluence, followed by transfection with 15 μg pCMV-VSV-G-A247, 7.5 μg pIRE4-Azi, 15 μg pCMV-capsid10-flag, and 15 μg pCMV-cargoCas9 by Polyjet according to the manufacturer’s instructions. The medium was supplemented with 1 mM Azi. The cell supernatant was collected twice at 48 h and at 72 h post-transfection. The supernatant was purified by centrifugation at 10000 × g for 1 h to remove cell debris and macro vesicles, followed by ultracentrifugation at 50000 g at 4°C for 2 h. The supernatant was decanted, and the remaining mBiosome^Cas9^ pellets were resuspended in PBS (pH 7.4) for further analysis.

For the preparation of reBiosome^αEmyc^, the above mBiosome^αEmyc^ suspensions were mixed with imidized DBCO-PEG solution at 37°C for 2 h. The resultant reBiosome^αEmyc^ was further purified by ultracentrifugation at 50000 g at 4°C for 2 h. The supernatant was decanted, and the remaining reBiosome^αEmyc^ pellets were resuspended in PBS (pH 7.4) for further analysis.

For the preparation of pegylated mBiosome^αEmyc^, the above mBiosome^αEmyc^ suspensions were mixed with DBCO-PEG solution at 37°C for 2 h. The resultant pegylated mBiosome^αEmyc^ was further purified by ultracentrifugation at 50000 g at 4°C for 2 h. The supernatant was decanted, and the remaining pegylated mBiosome^αEmyc^ pellets were resuspended in PBS (pH 7.4) for further analysis.

**Inhibitory effect of mBiosome^αEmyc^ on the proliferation of breast cancer cells**

MCF-7 cells (2 × 10^5^) were seeded in 6-well plates and transfected with mBiosome^αEmyc^ or mBiosome^Cas9^ (both packaging with 10 ng 5' frag) for 12 h. The culture medium was replaced with DMEM complete medium, and the cells were further incubated for 36 h. The cells were used to evaluate proliferation and apoptosis.

For the preparation of the proliferation of breast cancer cells, the viability of treated cells was measured at 0 h, 24 h, 48 h, 72 h and 96 h using a CCK-8 kit (Beyotime, Shanghai, China) according to the manufacturer’s instructions. The absorbance of each well was measured at 450 nm using a microplate reader (Infinite F50, Tecan Group, Beijing, China). For calculation of the relative cell viability, the cell viability at a fixed time point was normalized to that at 0 h.

For the evaluation of the apoptosis of breast cancer cells, the treated cells were tested by an Annexin V-FITC apoptosis assay kit according to the manufacturer’s instructions (#C1062L, Beyotime). Finally, the images were captured by fluorescence microscopy.

**Genomic PCR**

For investigation of αE_myc_ deletion status, MCF-7 cells (2 × 10^5^) were seeded in 6-well plates and transfected with mBiosome^αEmyc^ (packaging with 10 ng 5’ frag) for 12 h. Afterward, the culture medium was replaced by DMEM complete medium, and the cells were further incubated for 36 h. Finally, the cells were collected, and the genomic DNA was extracted from the cells by a tissue & cell genomic DNA purification kit (GeneMark, Beijing local agent, China). The concentration of genomic DNA was quantified with a Nano300 microspectrophotometer. The target genomic DNA sequences were amplified using I-5 2× high-fidelity master mix (Tsingke Biotechnology, Beijing, China) according to the manufacturer's instructions. All primer sets (Table S4, Supporting Information**)** were blasted by NCBI (National Center for Biotechnology Information, USA) to avoid amplification of nonspecific DNA. The PCR products were analyzed by agarose gel electrophoresis (1.5%, w/v) and confirmed by DNA sequencing (Tsingke Biotechnology, Beijing, China).

**qRT-PCR**

For analysis of the transcription level of c-Myc mRNA, MCF-7 cells (2 × 10^5^) were seeded in 6-well plates and transfected with mBiosome^αEmyc^ or mBiosome-Cas9 (both packaging with 10 ng 5' frag) for 12 h. Afterward, the culture medium was replaced by DMEM complete medium, and the cells were further incubated for 36 h. Finally, the cells were collected, and the total RNA was extracted from the cells using TRIzol (Invitrogen, Beijing local agent, China) according to the manufacturer’s instructions. The concentration of the total RNA was quantified with a Nano300 microspectrophotometer. cDNA was synthesized from the total RNA using PrimeScript RT reagent (TaKaRa, Shiga, Japan) by qRT-PCR (CFX connect system; Bio-Rad, CA, USA). Then, the cDNA was further amplified by adding specific primers for c-Myc mRNA (Table S4, Supporting Information) and SYBR Premix Ex Taq II (TaKaRa, Shiga, Japan) and measured by the same qRT–PCR system. The expression level of the c-Myc gene was normalized to that of GAPDH in each sample using the 2^−ΔΔCt^ method. Each experiment was performed in triplicate.

**Preparation of varying biosome^TNFα^**

pCMV-cargoshTNFα was constructed by cloning cargoshTNFα (shTNFα sequence grafted with 5' UTR, and 3' UTR of capsid 10) into the pCMV plasmid backbone. pCMV-cargoshcontrol was constructed by cloning cargoshcontrol (shcontrol sequence grafted with 5' UTR, and 3' UTR of capsid 10) into the pCMV plasmid backbone. The sequences of pCMV-cargoshTNFα and pCMV-cargoshcontrol were given in Dataset S1, and the sequences of shTNFα and shcontrol were given in Table S4, Supporting Information).

For the preparation of biosome packaging the TNFα gene silencing system (eBiosome^TNFα^ and mBiosome^TNFα^), HEK 293T cells were cultured in 225 cm^2^ flasks at 80-90% confluence, followed by transfection with 15 μg VSV-G encoding plasmid (pCMV-VSV-G or pCMV-VSV-G-A247), 7.5 μg pIRE4-Azi, 15 μg pCMV-capsid10-flag, and 15 μg pCMV-cargoshTNFα by Polyjet according to the manufacturer’s instructions. The medium was supplemented with 1 mM Azi. The cell supernatant was collected twice at 48 h and at 72 h post-transfection. The supernatant was purified by centrifugation at 10000 × g for 1 h to remove cell debris and macro vesicles, followed by ultracentrifugation at 50000 g at 4°C for 2 h. The supernatant was decanted, and the remaining mBiosome^TNFα^ pellets were resuspended in PBS (pH 7.4) for further analysis.

For the preparation of a mBiosome packaging nonspecific gene silencing system (mBiosome^con^), HEK 293T cells were cultured in 225 cm^2^ flasks at 80-90% confluence, followed by transfection with 15 μg pCMV-VSV-G-A247), 7.5 μg pIRE4-Azi, 15 μg pCMV-capsid10-flag, and 15 μg pCMV-cargoshcontrol by Polyjet according to the manufacturer’s instructions. The medium was supplemented with 1 mM Azi. The cell supernatant was collected twice at 48 h and at 72 h post-transfection. The supernatant was purified by centrifugation at 10000 × g for 1 h to remove cell debris and macro vesicles, followed by ultracentrifugation at 50000 g at 4°C for 2 h. The supernatant was decanted, and the remaining mBiosome^con^ pellets were resuspended in PBS (pH 7.4) for further analysis.

For the preparation of reBiosome^TNFα^, the above mBiosome^TNFα^ suspensions were mixed with imidized DBCO-PEG solution at 37°C for 2 h. The resultant reBiosome^TNFα^ was further purified by ultracentrifugation at 50000 g at 4°C for 2 h. The supernatant was decanted, and the remaining reBiosome^TNFα^ pellets were resuspended in PBS (pH 7.4) for further analysis.

For the preparation of pegylated mBiosome^TNFα^, the above mBiosome^TNFα^ suspensions were mixed with DBCO-PEG solution at 37°C for 2 h. The resultant pegylated mBiosome^TNFα^ was further purified by ultracentrifugation at 50000 g at 4°C for 2 h. The supernatant was decanted, and the remaining pegylated mBiosome^TNFα^ pellets were resuspended in PBS (pH 7.4) for further analysis.

**Inhibitory effect of mBiosome^TNFα^ on TNFα secretion by macrophages**

Murine macrophage RAW 264.7 cells (1× 10^4^) were seeded in 96-well plates and treated with 1 μg/mL lipopolysaccharide (LPS) under 5% CO_2_ at 37°C overnight. Then, the cells were transfected with mBiosome^TNFα^ or mBiosome^con^ (both packaging with 1 ng 5' frag) for 12 h. The culture medium was then replaced by DMEM complete medium, and the cells were further incubated for 36 h. Finally, the cell supernatant was collected to measure the concentration of TNFα by enzyme-linked immunosorbent assay (ELISA) according to the manufacturer’s instructions (# P06804, Cusabio, Wuhan, China).

**Polarization of macrophages by mBiosome^TNFα^**

Murine macrophage RAW 264.7 cells (1× 10^4^) were seeded in 96-well plates and treated with 1 μg/mL LPS under 5% CO_2_ at 37°C overnight. Then, the cells were transfected with mBiosome^TNFα^ or mBiosome^con^ (both packaging with 1 ng 5' frag) for 12 h. The culture medium was then replaced by DMEM complete medium, and the cells were further incubated for 36 h. The cells were collected and incubated with FITC anti-F4/80 antibody at 4°C for 30 min in the dark. Then, the cells were fixed with 4% polyformaldehyde for 10 min, followed by permeabilization with Triton-100 for 10 min. Afterward, the cells were incubated with APC anti-CD206 antibody at 4°C for 30 min in the dark. The cells were collected and rinsed twice with PBS (pH 7.4). Finally, FITC and APC intensities were analyzed at 488 nm and 638 nm, respectively, by FACScan flow cytometry. The F4/80-positive cells were selected to quantify the intensity of CD206 (a marker of macrophages), and the ratio of CD206^high^ cells was calculated by FlowJo software.

**Animals**

Female BALB/c nude mice (6 weeks; Charles River, Beijing, China) were used to establish the breast cancer-bearing model. Male DBA/1 mice (8 weeks, Charles River, Beijing, China) were used to establish collagen-induced arthritis (CIA) model. Female KM mice (8-10 weeks, Charles River, Beijing, China) were used to detect the immune response. The animals had free access to sterile food pellets and water, were kept in a specific pathogen-free (SPF) environment, and were kept under a 12 h light/dark cycle (temperature, 23 ± 2°C; relative humidity, 50 ± 20%). All animal experiments adhered to the principles of care and use of laboratory animals and were approved by the Institutional Animal Care and Use Committee of Peking University.

**Immunogenicity of biosomes in mice**

For analysis of the immunogenicity, saline (PBS pH 7.4), eBiosome^GFP^ and reBiosome^GFP^ were intravenously injected into KM female mice via the tail vein. Every single dose per mouse contained 100 ng 5' frag in biosome (based on qRT-PCR quantification). After dosing, the blood specimens were sampled at 6 h for routine blood tests and for virus-like immune response assays. To analyze the virus-like proinflammatory cytokines, the blood samples were centrifuged at 1500 × g for 20 min to collect the sera. The cytokines, consisting of interferons (IFN-α, IFN-β, IFN-γ), interleukins (IL-1β, IL-6, IL-10, IL-12), chemokines (CCL5, CXCL10, CCL2 and CXCL1), TNFα, and GM-CSF, were detected by a LEGENDplex^TM^ mouse anti-virus response panel (#740622, BioLegend, SA, USA) by following the kit instructions for flow cytometry (CytoFlex, Beckman Coulter, CA, USA). Data were analyzed by LEGENDplex data analysis software. Four cytokines (IL-6, IL-10, IL-12p70 and IFN-β) were not analyzed because they were below the limit of quantification.

**Pharmacokinetic study**

For the blood pharmacokinetics analysis, the reBiosome and the eBiosome were labeled with DiR by incubation at 25°C for 5 min, and intravenously injected into KM female mice via the tail vein, respectively. Every single dose per mouse contained 100 ng 5' frag in biosome (based on qRT-PCR quantification). After dosing, 10 μL blood specimens were collected by tail snip at 5 min, 15 min, 30 min, 1 h, 2 h, 4 h, 8 h, 12 h, 24 h, 36 h, and 48 h. The fluorescence intensity of DiR in the blood specimens was analyzed by a LivingImage system (PerkinElmer, MA, USA), and quantified by LivingImage software.

The areas under the fluorescence intensity-time curve (AUC) of each group were determined by the trapezoidal area method. The calibrated areas under the fluorescence intensity-time curve (ΔAUC) of reBiosome and eBiosome were obtained by subtracting the AUC of saline from the AUC of reBiosome and eBiosome, respectively.

**Breast cancer-bearing mice model**

For the establishment of the breast cancer-bearing mice model, human breast cancer MCF-7 cells (4 ×10^6^) were injected subcutaneously into the fourth mammary fat pad in the right of female BALB/c nude mice. The long diameter (L) and short diameter (S) of the tumor mass were recorded every other day. The tumor volume was calculated with the following formula: Volume (mm^3^) = 0.5 × L × S^2^. In addition, the body weight of each animal was monitored every other day.

**Anticancer efficacy of reBiosome^αEmyc^ in breast cancer-bearing mice**

The breast cancer-bearing mice were randomly divided into 4 groups (n=5 per group) when the tumor mass reached 50 mm^3^. At days 8, 10, 12 and 14, saline (PBS pH 7.4), eBiosome^αEmyc^, pegylated mBiosome^αEmyc^ and reBiosome^αEmyc^ were intravenously injected into the mice via the tail vein. Every single dose per mouse contained 100 ng 5' frag in biosome (based on qRT-PCR quantification). At day 16, the mice were sacrificed, and the tumor tissues were weighed. The tumor volume ratio was calculated by normalizing the tumor volume after treatment to that at day 8.

For measurement of c-Myc protein expression, cancer cells were isolated from tumor tissues by trypsinization after sacrificing the animals and lysed in RIPA buffer with protease phosphatase inhibitor. The cell lysate was centrifuged at 12000 rpm at 4°C for 20 min, and the total protein was collected from the supernatant and further analyzed by Western blots.

For the investigation of the histological changes, the main organs (heart, liver, spleen, lung, kidney) and tumor tissues were embedded in paraffin. The paraffin-embedded tissue sections were stained with a hematoxylin-eosin (HE) kit according to the manufacturer’s instructions and observed by a digital panoramic scanner (Wisleap, Jiangsu, China).

For the measurement of the expression of the proliferative antigen Ki67, paraffin-embedded tissue sections were deparaffinized in xylene, dehydrated in gradient ethanol (100% ethanol, 85% ethanol, and 75% ethanol for 5 min), and incubated with anti-Ki67 primary antibody at 4°C overnight, followed by incubation with HRP-conjugated secondary antibody at 25°C for 50 min. The sections were incubated with diaminobenzidine (DAB) chromogenic agent for approximately 30 s and incubated with hematoxylin stain solution for 3 min. The stained sections were observed by a multispectral tissue imaging system (Vectra Polaris, PerkinElmer, MA, USA). The Ki 67 expression of cancer cells was used to indicate the cancer proliferation, and Ki 67 positive cells (%) were quantified by Adobe Photoshop 2020.

For the evaluation of the apoptosis of tumor tissues, paraffin-embedded tissue sections were deparaffinized as described above and studied by terminal-deoxynucleotidyl transferase-mediated nick end labeling (TUNEL) assay according to the manufacturer’s instructions (#GDP1042, Servicebio, Wuhan, China). Briefly, the sections were incubated with terminal deoxynucleotidyl transferase (TDT) enzyme, FITC-dUTP and equilibration buffer (1:5:50, v/v/v) at 37°C for 2 h, followed by incubation with DAPI solution at 25°C for 10 min in the dark. The stained sections were observed by a multispectral tissue imaging system (Vectra Polaris). The green dots labeled with FITC-dUTP were used to indicate apoptotic cells, and apoptotic cells (%) were quantified by Adobe Photoshop 2020.

**Collagen-induced arthritis mice model**

For the establishment of collagen-induced arthritis (CIA) mouse model, 0.1 mL emulsions consisting of type II bovine collagen and CFA (1:1, v/v) were injected subcutaneously into a male DBA/1 mouse at the base of the tail at day 0, followed by a booster of 0.1 mL emulsions consisting of type II bovine collagen and IFA (1:1, v/v) at the same location at day 21. The joint inflammation signs of the mice were recorded every other day after the booster, consisting of the following: 0, normal; 1, erythema and mild swelling confined to the ankle joint; 2, erythema and mild swelling extending from the ankle to metatarsal or metacarpal joints; 3, erythema and moderate swelling extending from the ankle to the metatarsophalangeal or metacarpophalangeal joints; 4, erythema and severe swelling extending from the ankle to the digits [8]. Each paw was scored from 0 to 4, and then, the scores were summed to give each mouse an arthritis score. In addition to the arthritis score, each mouse's weight and the thickness of four paws were measured every other day.

**Anti-inflammatory efficacy of reBiosome^TNFα^ in CIA mice**

The CIA mice were randomly divided into 4 groups (n=5 per group). At days 25, 27, 29 and 31, saline (PBS pH 7.4), eBiosome^TNFα^, pegylated mBiosome^TNFα^ or reBiosome^TNFα^ was injected intravenously into the CIA mice via the tail vein. Every single dose per mouse contained 100 ng 5' frag in biosome (based on qRT-PCR quantification). At day 33, the mice were sacrificed to collect blood, major organs (heart, liver, spleen, lung, kidney) and arthritic joints (knee and ankle). The blood samples were used to measure the level of TNFα by ELISA as described above. The ankle joints were fixed with 4% paraformaldehyde and decalcified with 10% EDTA-2Na solution, followed by embedding with paraffin.

For the observation of histological inflammation in knee joints, paraffin-embedded tissue sections were stained with HE and then with safranin and fast green (SF) according to the manufacturer’s instructions. The stained sections were observed by a digital panoramic scanner (WISLEAP, Jiangsu, China).

For the evaluation of the polarization of macrophages, the paraffin-embedded sections were deparaffinized as described above and incubated with anti-F4/80 primary antibody at 4°C overnight, followed by incubation with FITC-conjugated secondary antibody at 25°C for 50 min in the dark. The sections were incubated with anti-CD206 primary antibody at 4°C overnight, followed by incubation with the Cy5-conjugated secondary antibody at 25°C for 50 min in the dark. Afterward, the sections were incubated with DAPI solution at 25°C for 10 min in the dark and observed by a multispectral tissue imaging system (Vectra Polaris).

For the evaluation of the expression of TNFα, the paraffin-embedded sections were deparaffinized as described above and incubated with anti-TNFα primary antibody at 4°C overnight, followed by incubation with FITC-conjugated secondary antibody at 25°C for 50 min in the dark. Afterward, the sections were incubated with DAPI (4',6-diamidino-2-phenylindole) solution at 25°C for 10 min in the dark and observed by a multispectral tissue imaging system (Vectra Polaris).

**Micro-CT analysis**

For the observation of osteolysis in the paws of the mice with arthritis, the left hind paws of CIA DBA/1 mice were scanned by micro-CT (X-ray microtomography, Skyscan, Bruker, Germany). The scanning parameters consisted of the following: voltage, 60 kV; current, 200 μA; and resolution, 6.5 μm. Three-dimensional images were reconstructed using N-Recon software (Skyscan, Bruker, Germany).

**Imaging distribution of varying biosome^GFP^ in mice**

For the analysis of the distribution in breast cancer-bearing mice, varying biosomes packaging the GFP expressing system (eBiosome^GFP^, pegylated mBiosome^GFP^, reBiosome^GFP^) were labeled with DiR by incubation at 25°C for 5 min. The breast cancer-bearing mice and divided into 4 groups (n=3 per group) at day 10. Saline (PBS pH 7.4) and varying DiR-labeled biosome (eBiosome^GFP^, pegylated mBiosome^GFP^, and reBiosome^GFP^) were intravenously injected into breast cancer-bearing mice via the tail vein. Every single dose per mouse contained 100 ng 5' frag in biosome (based on qRT-PCR quantification). The mice were sacrificed at 96 h, and the main organs (heart, liver, spleen, lung, and kidney) and tumor tissues were collected to measure the fluorescence intensity of DiR and GFP by a LivingImage system (PerkinElmer, MA, USA). The DiR and GFP fluorescence intensity in organs and in tumor tissues was quantified by LivingImage software.

For the analysis of the distribution in CIA mice, the mice with arthritis were divided into 4 groups (n=3 per group) at day 26, and were treated varying biosomes prepared as above mentioned. The mice were sacrificed at 96 h, and the main organs (heart, liver, spleen, lung, and kidney) and arthritic tissues were collected to measure the fluorescence intensity of DiR and GFP by a LivingImage system (PerkinElmer, MA, USA). The DiR and GFP fluorescence intensity in organs and in arthritic tissues was quantified by LivingImage software.

**Statistical analysis**

Prism 7 (GraphPad, La Jolla, CA, USA) was used for statistical analyses, unless otherwise specified. The two-tailed unpaired Student's t test was used to determine the significance between groups, and two-way analysis of variance (ANOVA) was used to determine the significance among groups of two categorical variables. The results are presented as the mean ± standard deviation. A value of p<0.05 was considered statistically significant.

**Supplementary Results**

**Synthesis and characterization of** **stealth material**

For synthesis of the stealth material, polyethylene glycol aldehyde (mPEG-CHO, 2000 Da) was reacted with dibenzocyclooxtyne amine (DBCO-NH_2_) to form imidized DBCO-PEG by dehydration under pyrrolidine catalysis (Fig. S16a). The results from mass spectrometry (MALDI-TOF MS) showed that the molecular weight of imidized DBCO-PEG conformed to the formula MW = 44n + 408 (Fig. S16b). The results from ^1^H nuclear magnetic resonance (NMR) spectrometry showed that the characteristic peak of aldehyde hydrogen (a) in PEG-CHO disappeared while the characteristic peaks of aliphatic hydrogens (b-d) in mPEG-CHO, the characteristic peaks of aromatic hydrogens (f) in DBCO-NH_2_ and the characteristic peak of imine hydrogen (m) were found in the ^1^H NMR spectrum of imidized DBCO-PEG (Fig. S17). Moreover, the results from ^13^C nuclear magnetic resonance (NMR) showed that the chemical shift of carbonyl carbon (a) in imidized DBCO-PEG was reduced compared to that in mPEG-CHO while the characteristic peaks of methylene carbons (b-c) in mPEG-CHO and aromatic carbons (f) in DBCO-NH_2_ were observed in the ^13^C NMR spectrum of imidized DBCO-PEG (Fig. S18). In addition, the results from infrared (IR) spectrometry showed that the rocking vibration C-H bond peak of methylene (841 cm^-1^) and the out-of-plane bending vibration peaks of benzene (801 cm^-1^) and imine (697 cm^-1^) were observed in the IR spectrum of imidized DBCO-PEG (Fig. S16c). Accordingly, these measurements demonstrated that the imidized DBCO-PEG was successfully synthesized.

For evaluation of the hydrolytic efficiency of imidized DBCO-PEG in a weakly acidic environment, a high-performance liquid chromatography (HPLC) method was established for quantifying DBCO-NH_2_ (Fig. S16d), and the hydrolytic study of imidized DBCO-PEG was performed in phosphate-buffered saline (PBS pH 7.4, pH 6.5 and pH 5.0). The results showed that the imidized DBCO-PEG was readily hydrolyzed in weakly acidic media (hydrolysis 55% in pH 5.0 PBS at 12 h and 28% in pH 6.5 PBS at 12 h) compared with that in physiological medium (hydrolysis 5% pH 7.4 PBS at 12 h) (Fig. S16e).

**Supplementary figures**


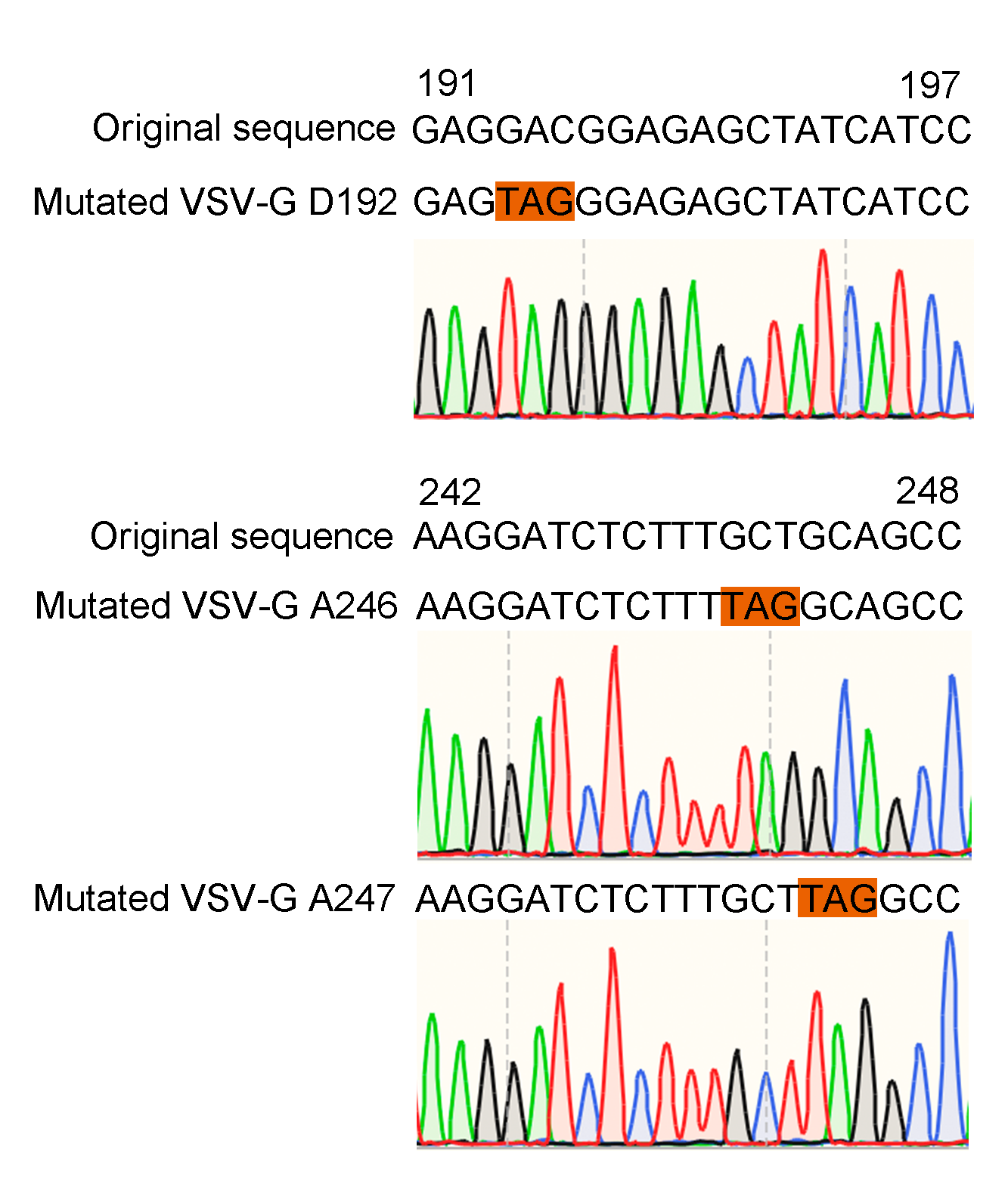


**Fig. S1** Verification of the site-specific mutations in VSV-G-expressing plasmids by DNA sequencing. The sequences of varying mutated VSV-G-expressing plasmids (pCMV-VSV-G-D192, pCMV-VSV-G-A246 and pCMV-VSV-G-A247) were aligned to the unmutated VSV-G-expressing plasmid pCMV-VSV-G. At the D192, A246, or A247 site, the original codon was mutated to the blank codon TAG, highlighted by a red rectangle, respectively.


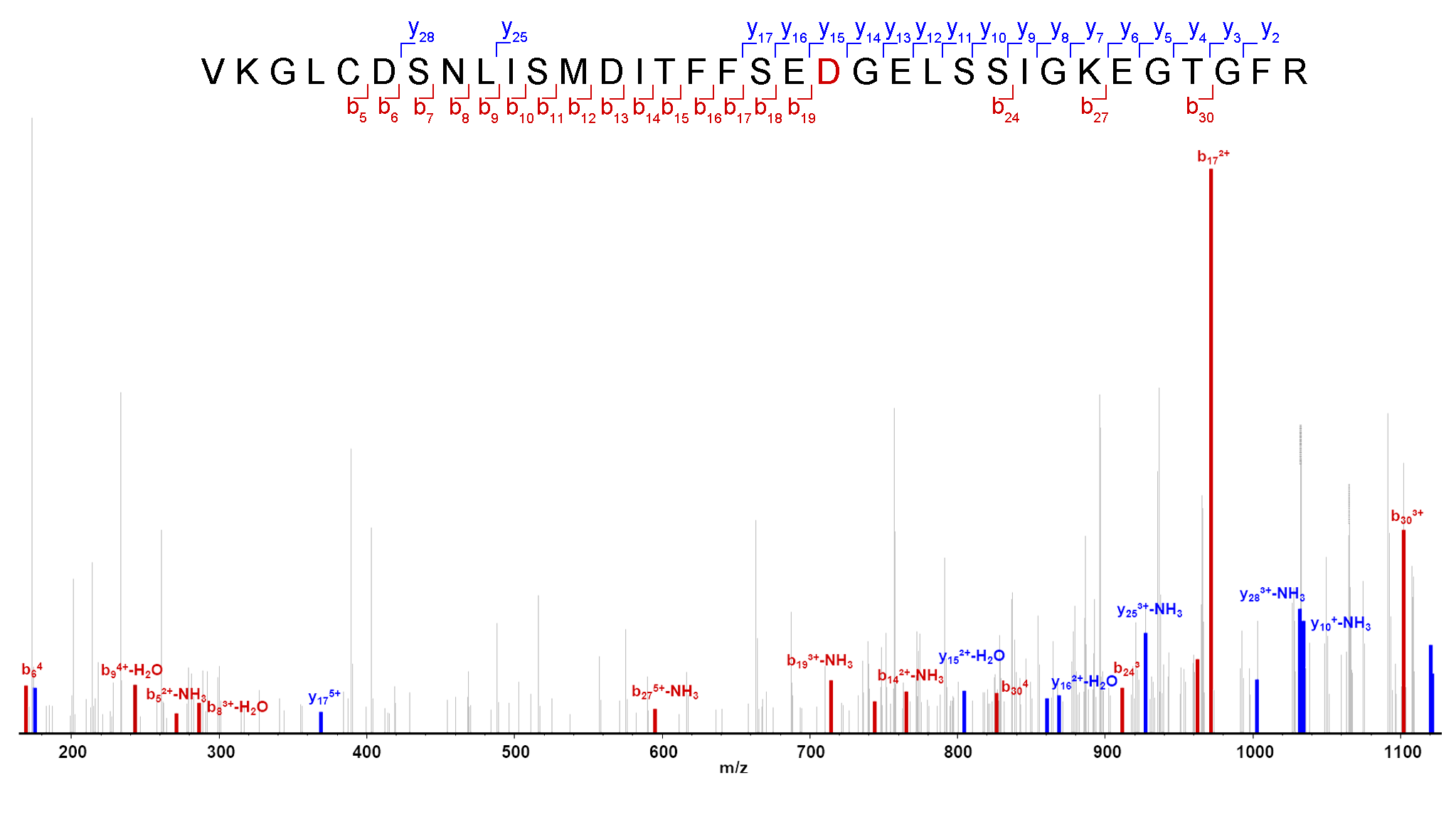


**Fig. S2** Site-specific display of Azi on mProtein by peptide sequencing. The site-specific display of Azi at site 192 in mProtein D192, as a typical site, was verified by LC-MS/MS spectrum of a peptide derived from the purified protein after trypsinization.


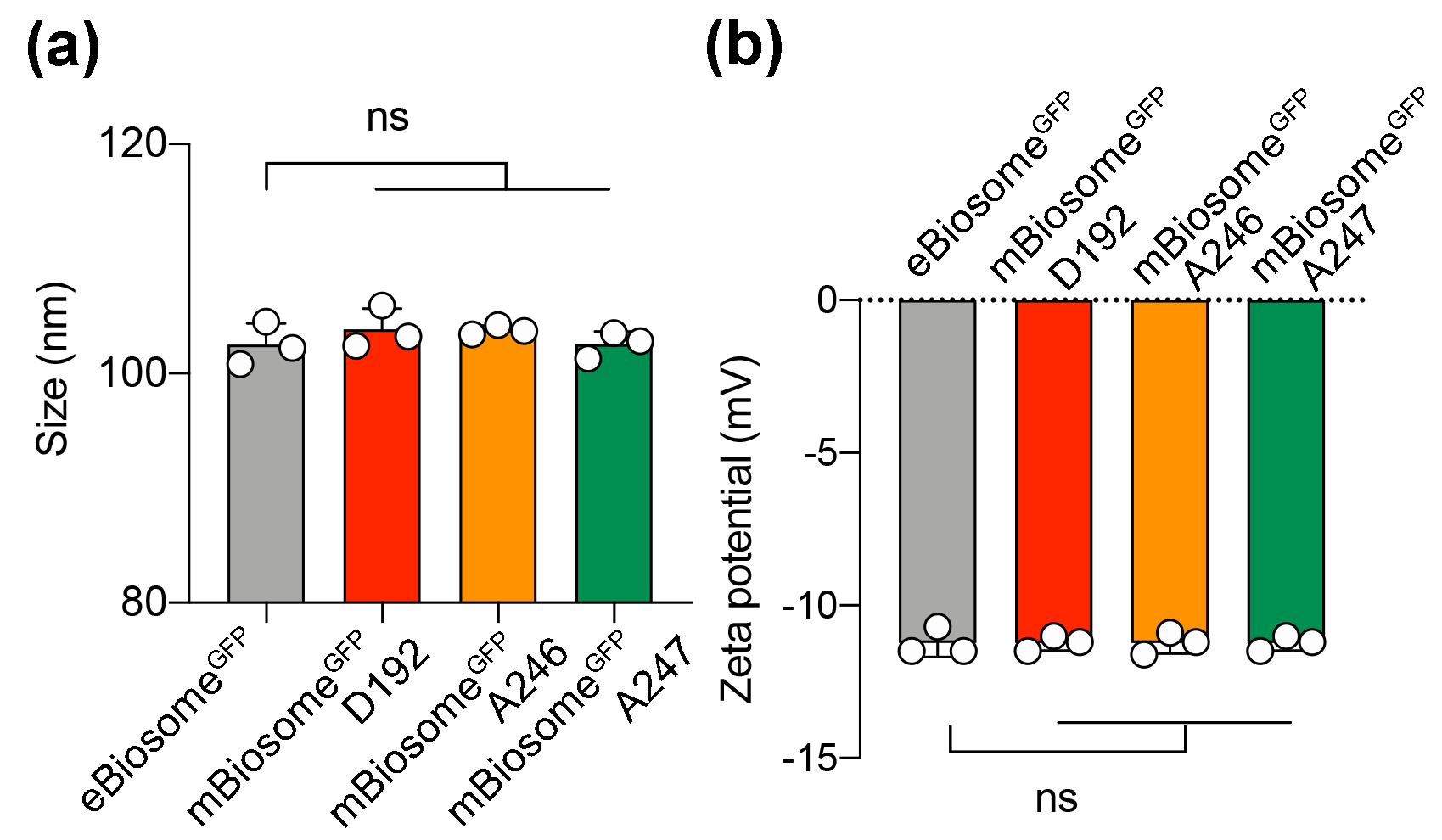


**Fig. S3** Particle sizes and Zeta potential values of mBiosomes^GFP^. **a** Average sizes of mBiosomes^GFP^. **b** Zeta potential values of mBiosomes^GFP^. The study was performed by dynamic light scattering (DLS).


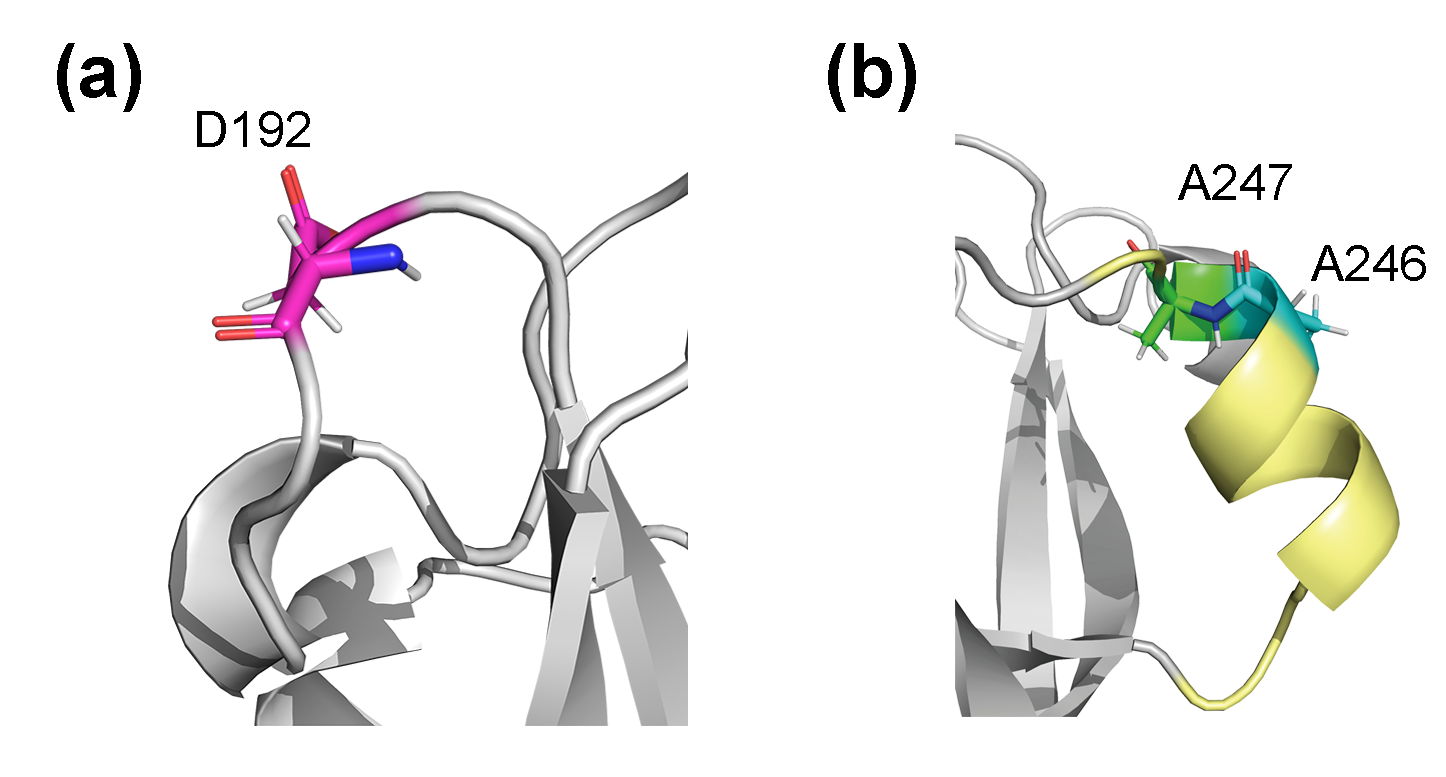


**Fig. S4** Illustration for the surrounding secondary structures of mutation sites. **a** Site 192 (purple) displayed with Azi. **b** Site 246 (blue) and site 247 (green) displayed with Azi.


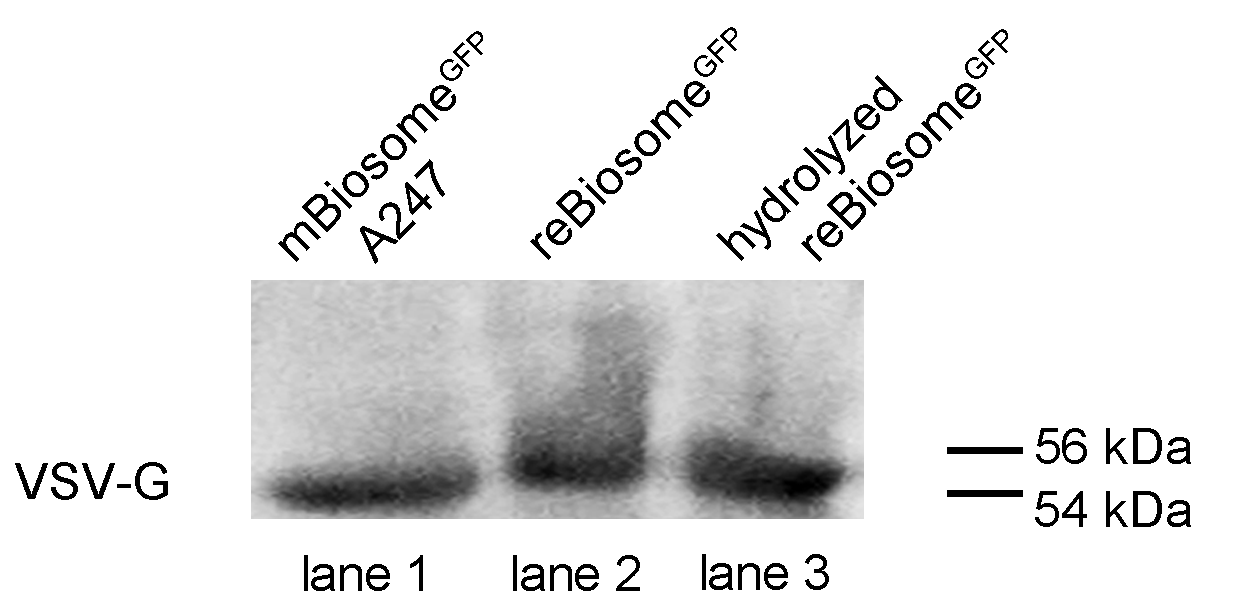


**Fig.S5** Molecular weight of VSV-G in reBiosome^GFP^ and hydrolyzed reBiosome^GFP^ were compared with that in mBiosome^GFP^ A247. The study was performed by Western blot.


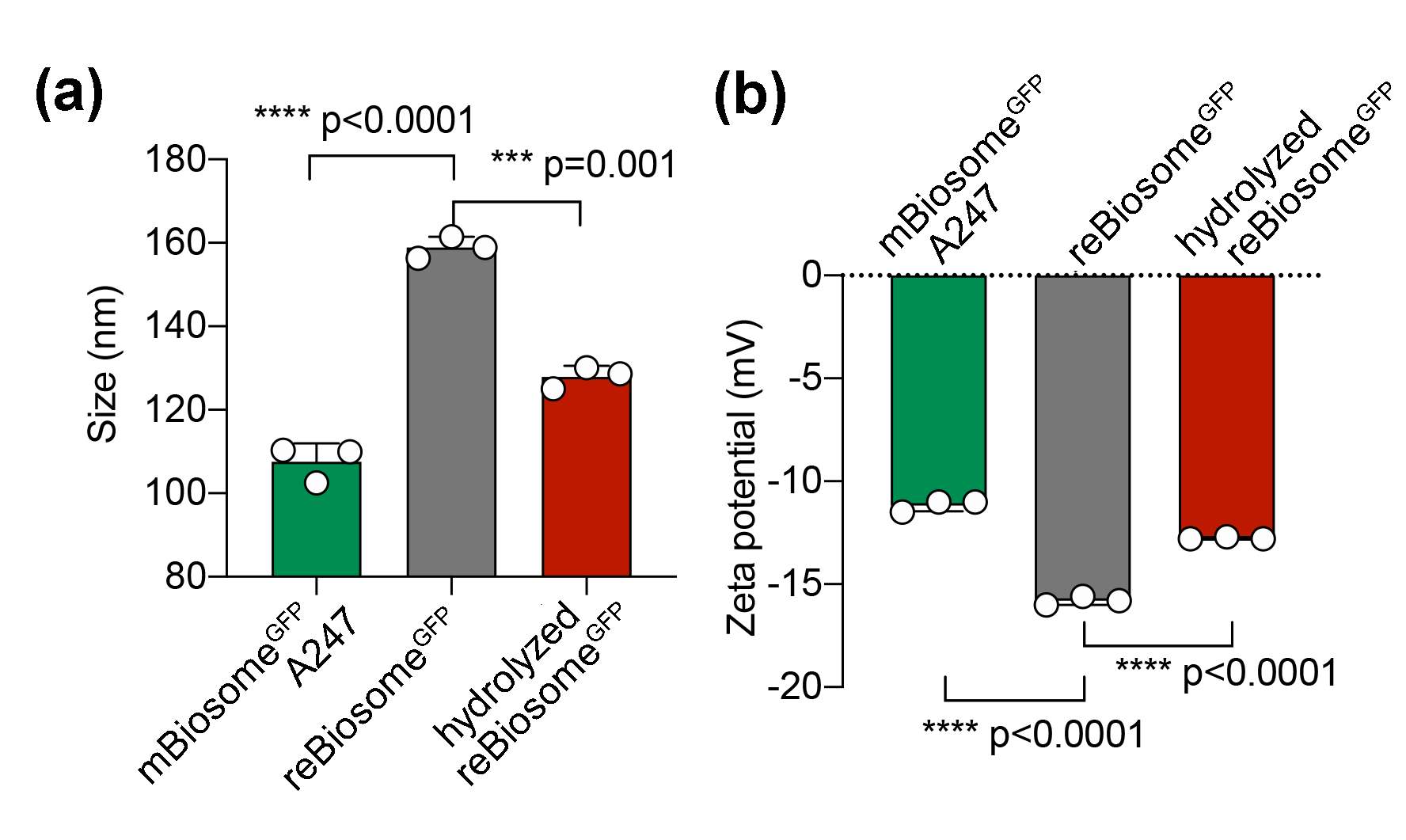


**Fig. S6** **a** Average sizes of reBiosome^GFP^ and hydrolyzed reBiosome^GFP^. **b** Zeta potential values of mBiosomes^GFP^ hydrolyzed reBiosome^GFP^. Studies were performed by dynamic light scattering (DLS).


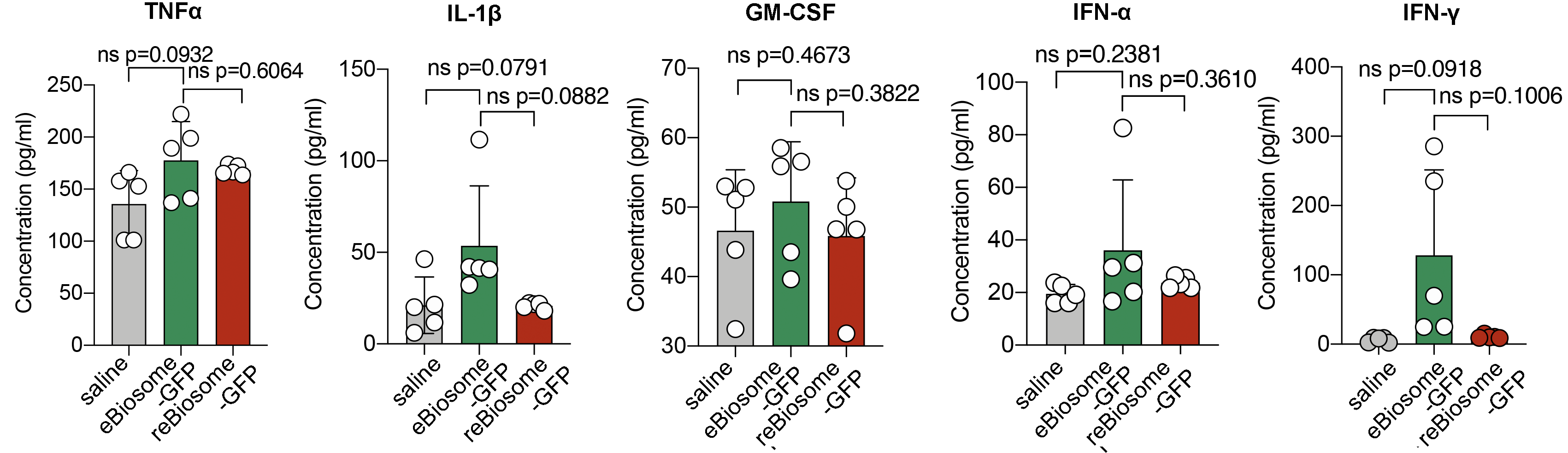


**Fig. S7** Quantification of concentrations of TNFα, IL-1β, GM-CSF, IFN-α and IFN-γ. Data are represented as the mean ± standard deviation (n=5). The two-tailed unpaired Student's t-test was used to determine the significance between groups.


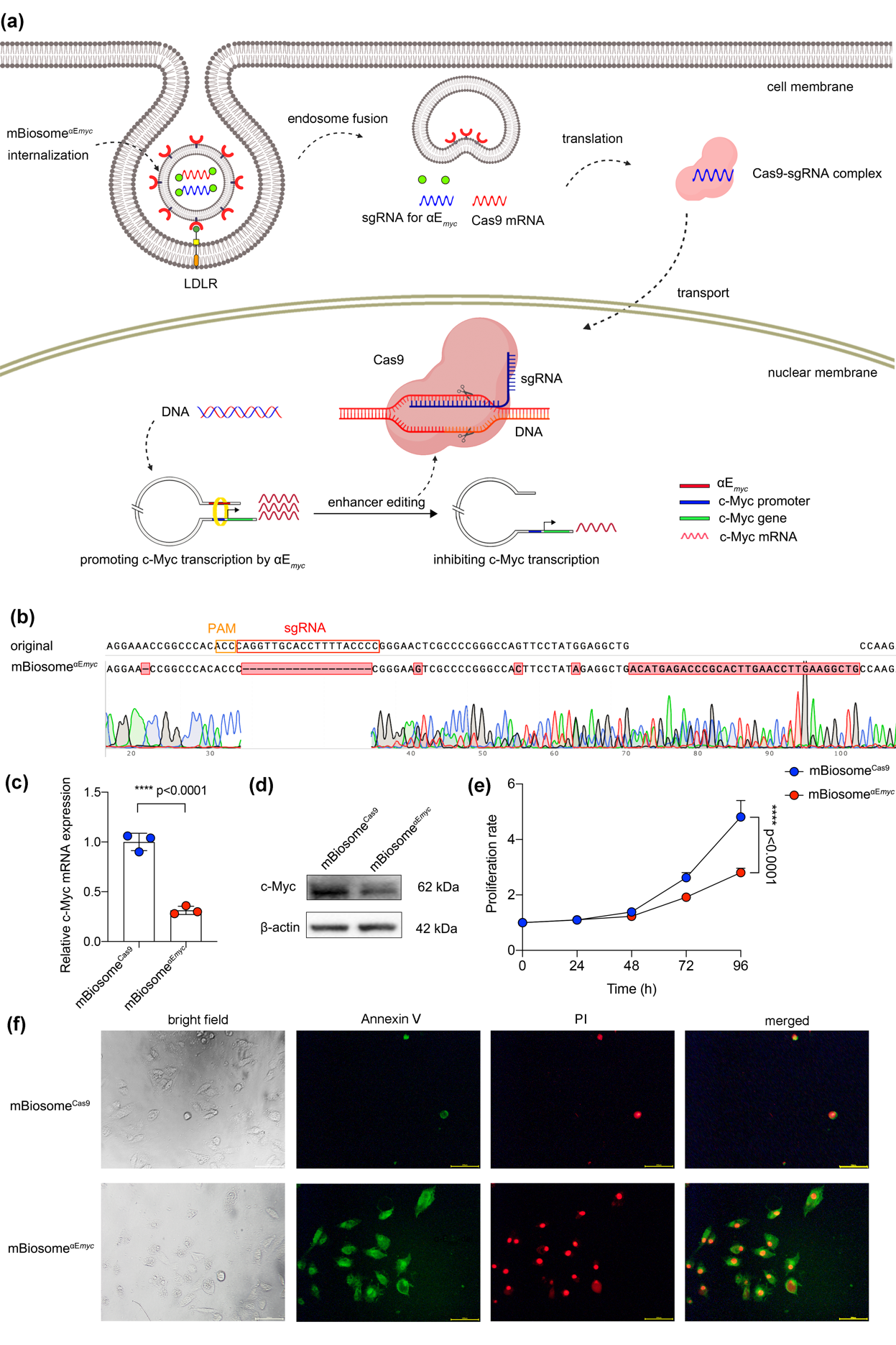


**Fig. S8** Inhibitory effect of mBiosome^αE^*^myc^* in breast cancer cells. **a** Illustration for the reduced c-Myc mRNA transcription by treatment with mBiosome^αE^*^myc^*. The mBiosome^αE^*^myc^* was internalized by breast cancer MCF-7 cells mediated by LDLR, and then, the αE*_myc_* gene editing system (including Cas9 mRNA and sgRNA specific for αE*_myc_*) was released into the cytoplasm by endosome fusion. Cas9 mRNA was translated into Cas9 protein, which formed a complex with sgRNA. Then, the complex entered the nucleus and further deleted the oncogenic enhancer (αE*_myc_*) sequence, thereby leading to the inhibition of the c-Myc transcription process. **b** Deleted αE*_myc_* DNA sequence by treatment with mBiosome^αE^*^myc^* in breast cancer cells. Genomic DNA was extracted from the mBiosome^αE^*^myc^*-treated cells for DNA sequencing. The DNA sequence of the mBiosome^αE^*^myc^*-treated cells was aligned to the sequence of the normal MCF-7 cells. **c** Reduced c-Myc mRNA transcription by treatment with mBiosome^αE^*^myc^* in breast cancer cells. Total mRNA was extracted from mBiosome^αE^*^myc^*-treated or mBiosome^Cas9^-treated cells for qRT-PCR analysis. **d** Decreased c-Myc protein expression by treatment with mBiosome^αE^*^myc^* in breast cancer cells. Total protein was extracted from mBiosome^αE^*^myc^*-treated cells or mBiosome^Cas9^-treated cells for Western blot analysis. β-actin was used as the internal reference. **e** Proliferation inhibited by treatment with mBiosome^αE^*^myc^* in breast cancer cells. The proliferation rates of mBiosome^αE^*^myc^*-treated cells and mBiosome^Cas9^-treated cells were measured by CCK-8 assays at fixed time points. **f** Apoptosis induced by treatment with mBiosome^αE^*^myc^* in breast cancer cells. The apoptosis of mBiosome^αE^*^myc^*-treated cells and mBiosome^Cas9^-treated cells was measured by an Annexin V-FITC/PI assay, and representative images captured by fluorescence microscopy were presented. Green channel, Annexin V stained by FITC; red channel, nuclei stained by PI; merged, a combination of the above channels. Scale bar, 200 μm. Data are represented as the mean ± standard deviation (n=3). In Fig. S8c, the two-tailed unpaired Student's t test was used to determine the significance between groups. In Fig. S8e, the two-way analysis of variance (ANOVA) was used to determine the significance among groups.


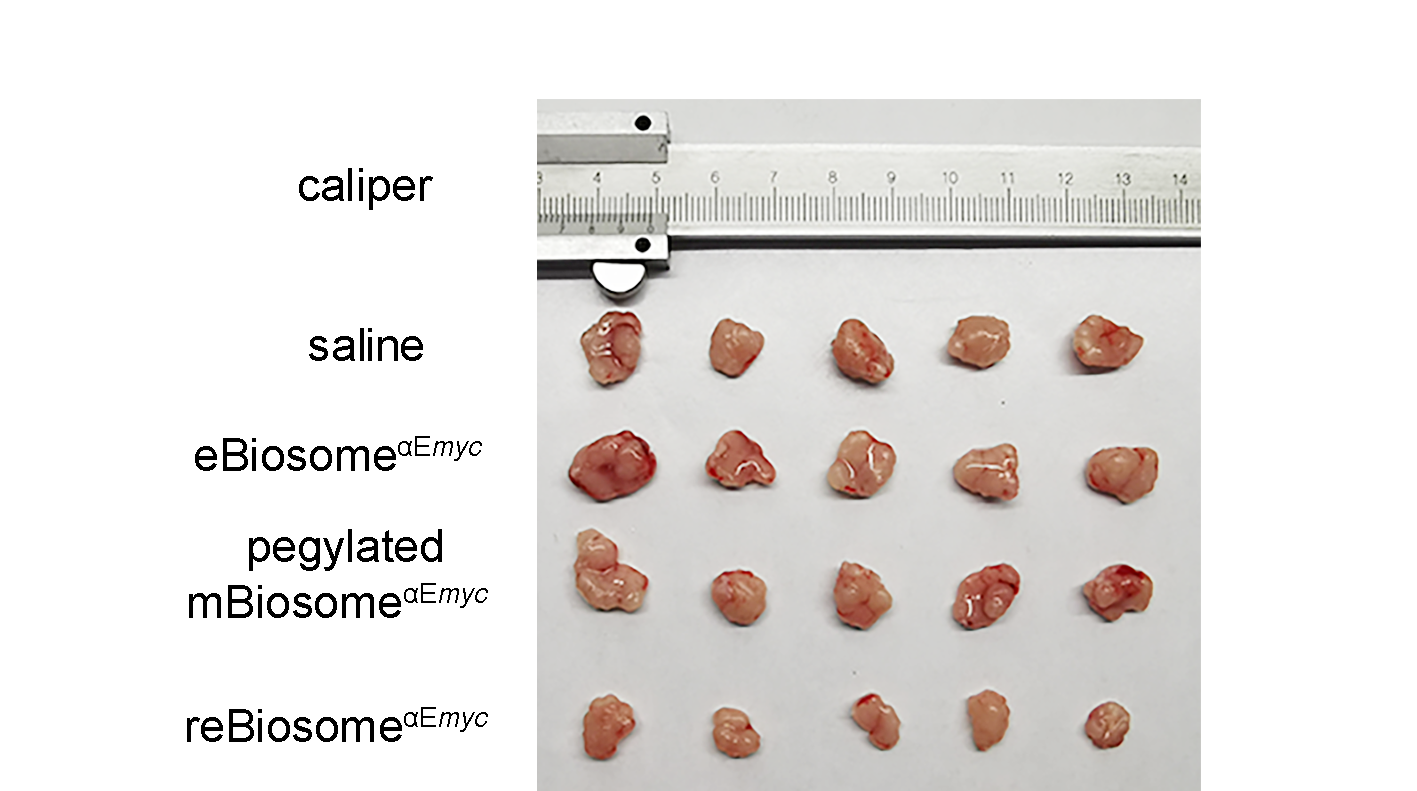


**Fig. S9** Inhibitory effect of reBiosome^αE^*^myc^* on the growth of tumor tissues in breast cancer-bearing mice. At day 16, the mice were sacrificed, and the tumor tissues were photographed.


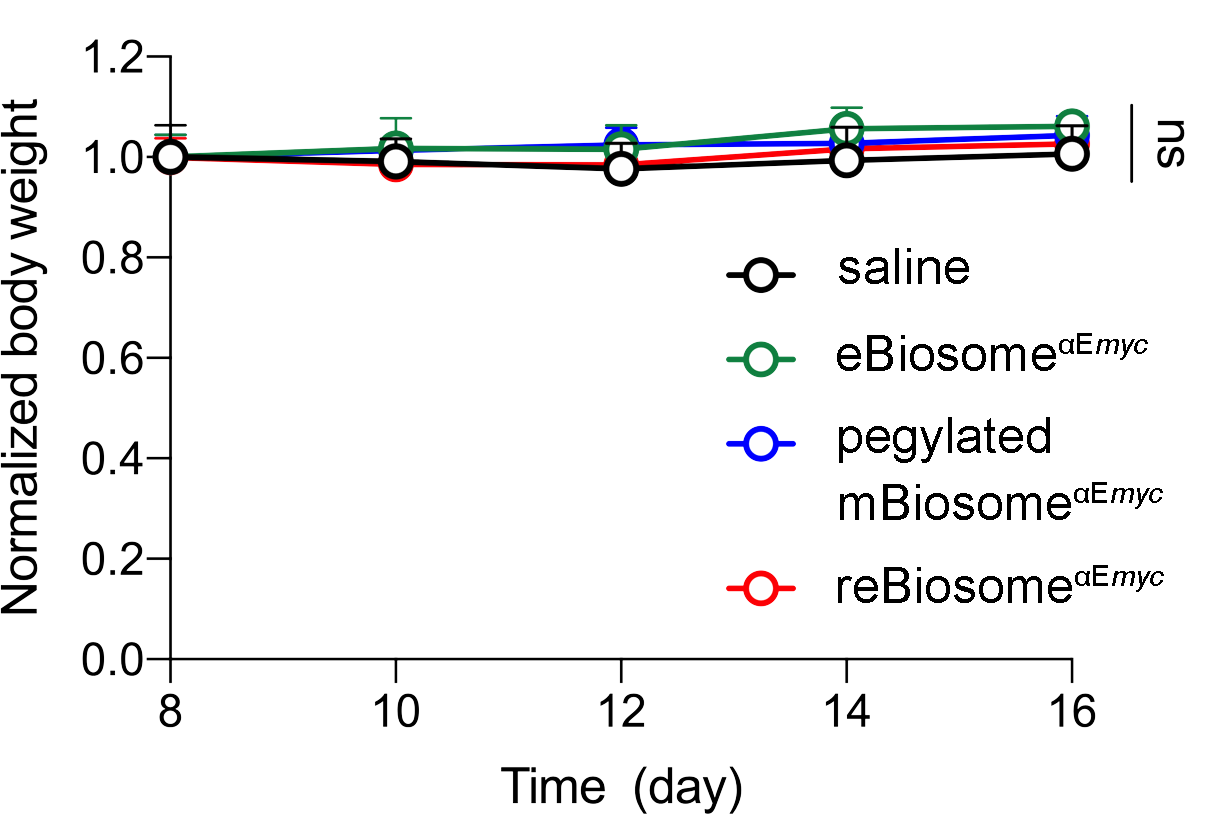


**Fig. S10** Effect of reBiosome^αE^*^myc^* on the body weights of breast cancer-bearing mice. The body weight of each animal was normalized to that at day 8. Data are represented as the mean ± standard deviation (n=5). The two-way analysis of variance (ANOVA) was used to determine the significance among groups.


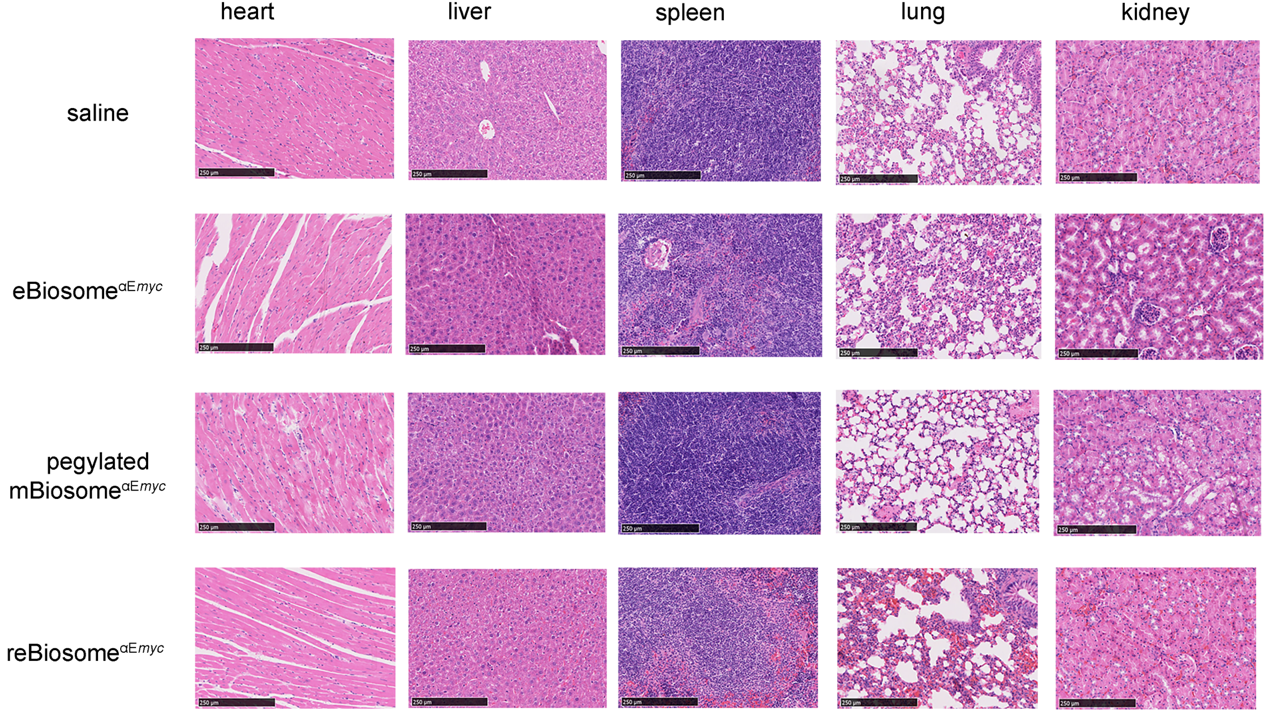


**Fig. S11** Effect of reBiosome^αE^*^myc^* on the major organs of breast cancer-bearing mice. At day 16, the major organs (heart, liver, spleen, lung and kidney) of breast cancer-bearing mice were collected after sacrificing the animals and stained with HE to evaluate the effects. Scale bar, 250 μm.


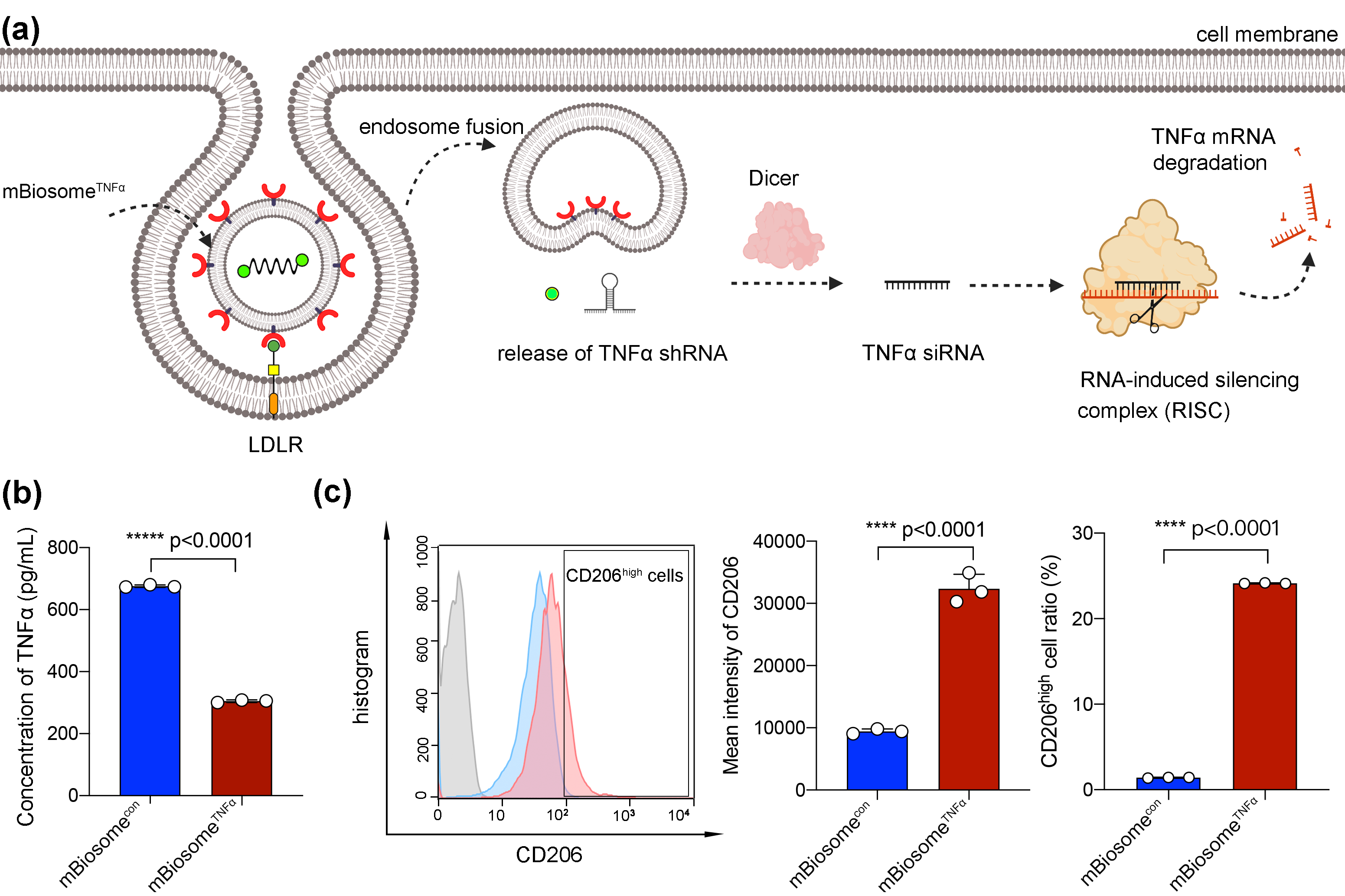


**Fig. S12** TNFα gene silencing and polarization by mBiosome^TNFα^ in macrophages.

**a** Illustration for reduced TNFα expression in macrophages by treatment with mBiosome^TNFα^. The mBiosome^TNFα^ was internalized by macrophages (RAW 264.7 cells) mediated by LDLR, and then, the TNFα gene silencing system (shRNA for TNFα) was released into the cytoplasm by endosome fusion. The shRNA was digested into siRNA by Dicer, further forming the RNA-induced silencing complex (RISC). Afterward, TNFα mRNA was degraded by RISC, leading to the inhibition of TNFα protein translation. **b** Decreased production of TNFα by mBiosome^TNFα^ in macrophages. The supernatants of RAW 264.7 cells transfected with mBiosome^TNFα^ and mBiosome^con^ were collected to measure the concentration of TNFα using a TNFα ELISA kit. **c** Induced polarization by mBiosome^TNFα^ in macrophages. **Left:** histogram of CD206 intensity. Gray, blank control; blue, mBiosome^con^; red, mBiosome^TNFα^ treatment. **Middle:** quantification of CD206 fluorescence intensity. **Right:** quantification of the CD206^high^ cell ratio. Data are represented as the mean ± standard deviation (n=3). The two-tailed unpaired Student's t-test was used to determine the significance between groups.


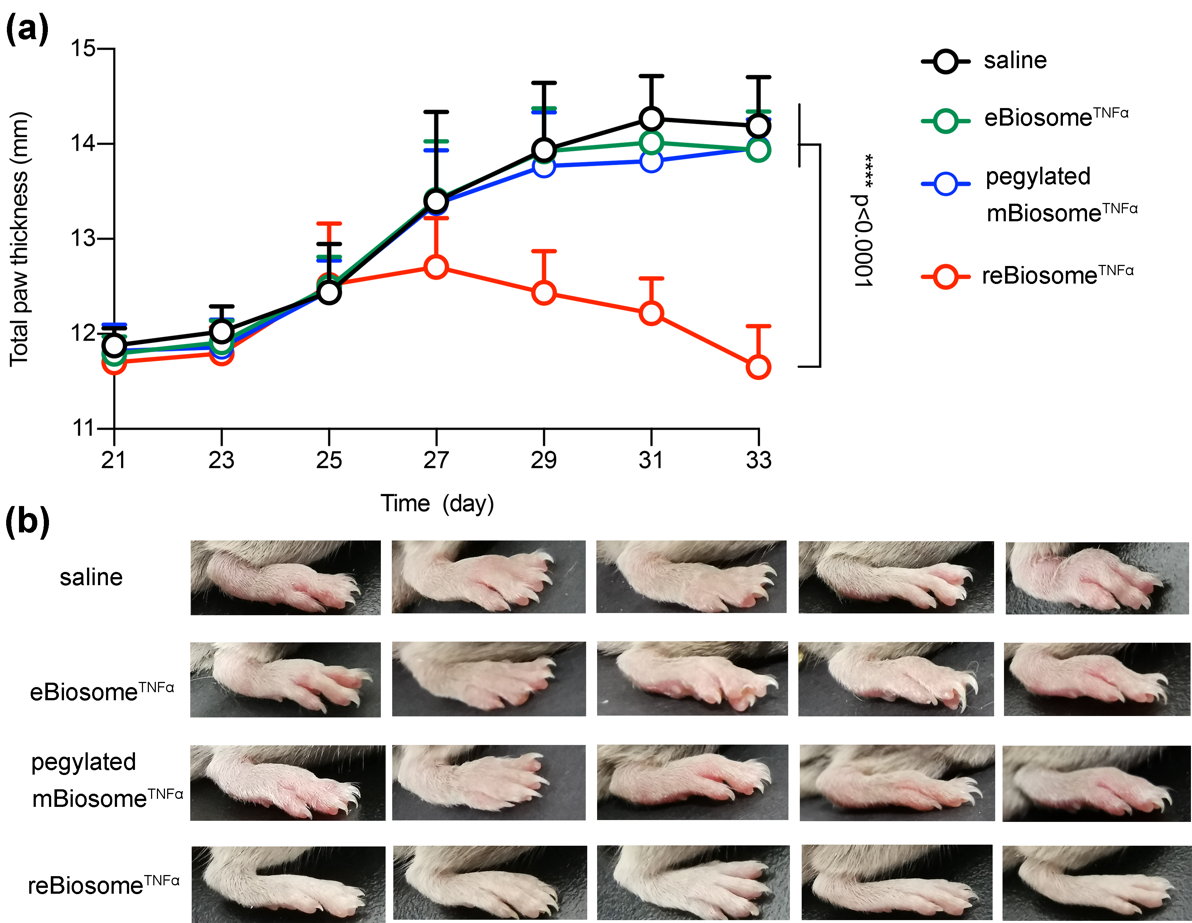


**Fig. S13** Inhibitory effect of reBiosome^TNFα^ on inflammation in CIA mice. **a** Inhibited inflammation by reBiosome^TNFα^ treatment on paw thickness of CIA mice. The paw thickness of CIA mice was recorded every other day after the booster at day 21. The total paw thickness was calculated by the thickness of the four paws. **b** Inhibited inflammation by reBiosome^TNFα^ treatment on paw swelling of CIA mice. At day 33, the left hind paws of the mice were photographed. Data are represented as the mean ± standard deviation (n=5). The two-way analysis of variance (ANOVA) was used to determine the significance among groups.


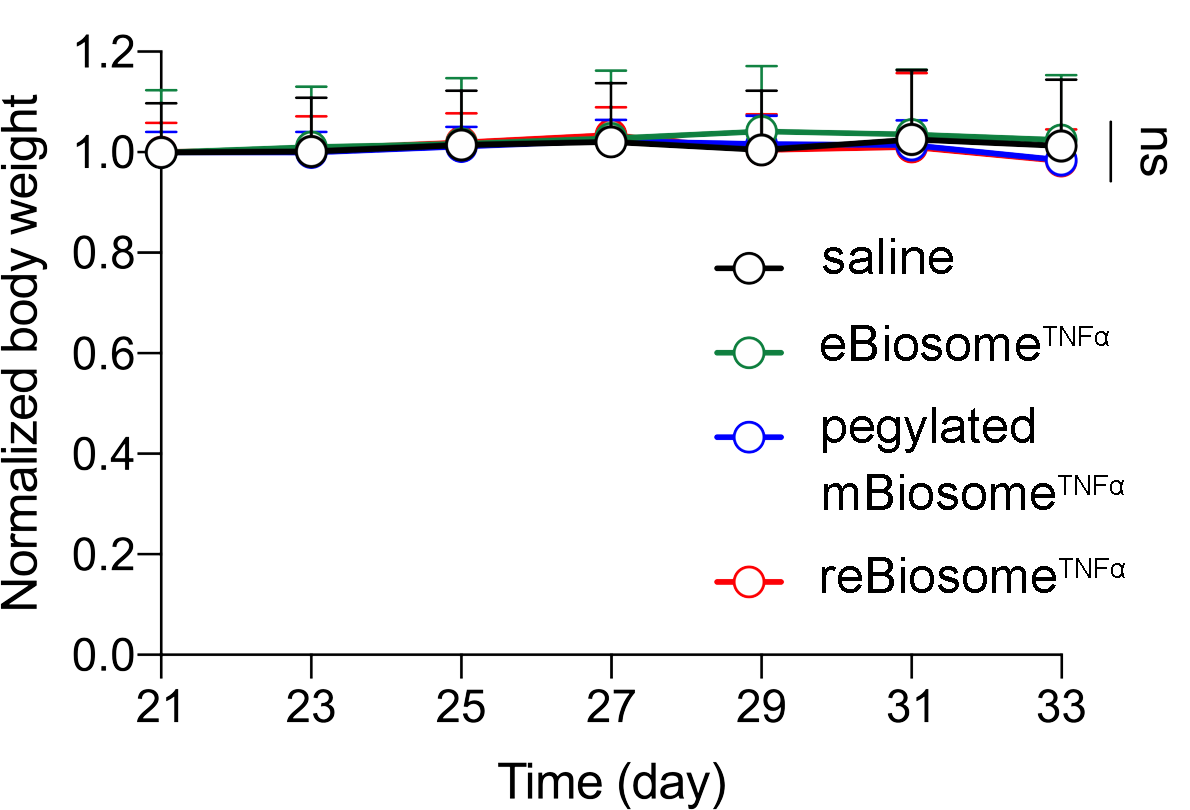


**Fig. S14** Effect of reBiosome^TNFα^ on the body weights of CIA mice. The body weight of each animal was normalized to that at day 21. Data are represented as the mean ± standard deviation (n=5). The two-way analysis of variance (ANOVA) was used to determine the significance among groups.


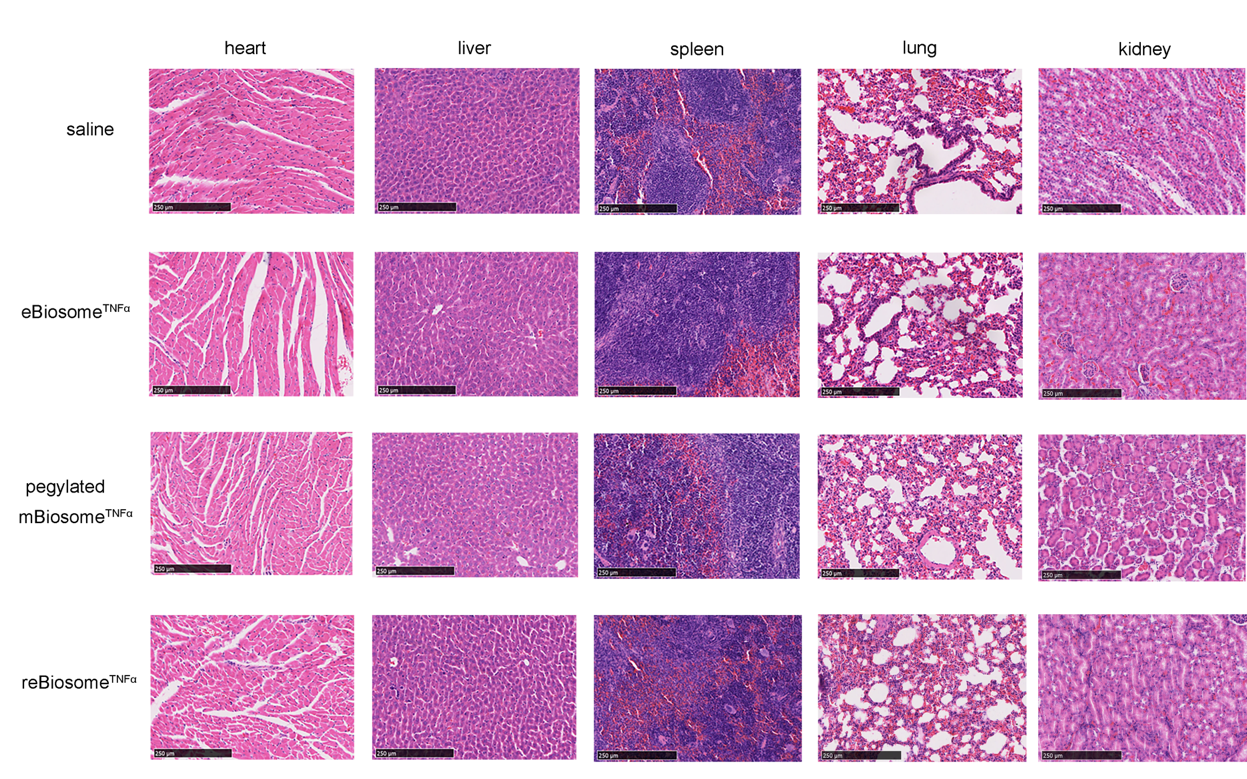


**Fig. S15** Effect of reBiosome^TNFα^ on the major organs of CIA mice. At day 33, the major organs (heart, liver, spleen, lung, and kidney) of CIA mice were collected after sacrificing the animals and stained with HE to evaluate the effects. Scale bar, 250 μm.


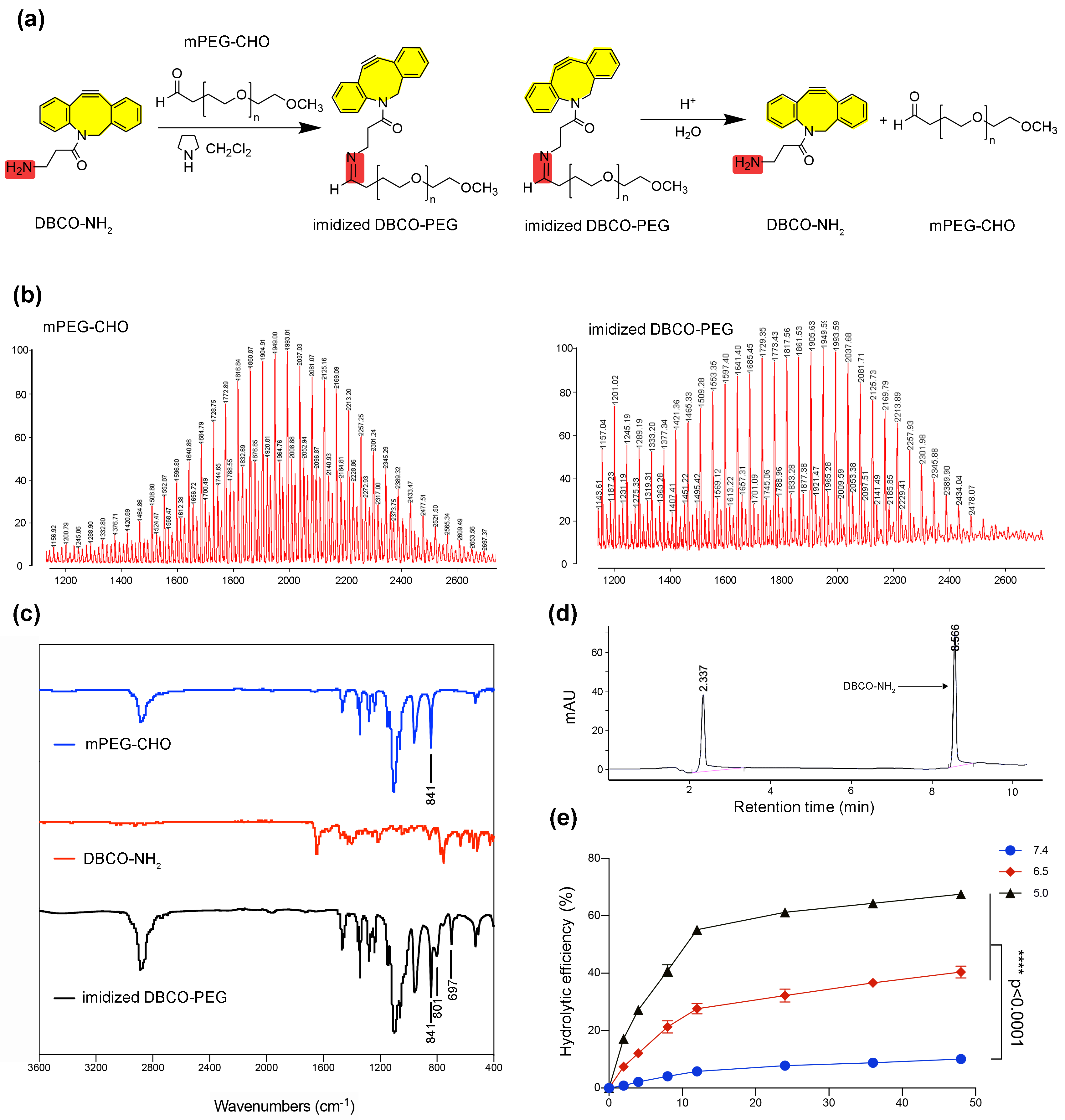


**Fig. S16** Synthesis and characterization of the stealth material polyethylene glycol derivative. **a** Schemes for the synthetic process and hydrolytic process of imidized DBCO-PEG. Imidized DBCO-PEG (mPEG-CH=N-DBCO) was prepared by reacting DBCO-NH_2_ with mPEG-CHO via dehydration. Under a weakly acidic environment, imidized DBCO-PEG was hydrolyzed to form DBCO-NH_2_ and mPEG-CHO. **b** Time-of-flight mass spectra (TOF MS) of mPEG-CHO and imidized DBCO-PEG. Distributions of the molecular weight for mPEG-CHO (left) and imidized DBCO-PEG (right) were assigned to the characteristic peaks of PEG derivative polymers. **c** Infrared spectra of mPEG-CHO (blue), DBCO-NH_2_ (red) and imidized DBCO-PEG (black). Each characteristic peak was assigned to its chemical structure, as labeled on the spectra. **d** Typical HPLC chromatogram of DBCO-NH_2_. HPLC analysis was performed on a C18 column with a UV detector at 25°C. The mobile phase consisted of methanol and water (70:30, v/v). The detection wavelength was set at 210 nm, and the flow rate was 0.5 mL/min. DBCO-NH_2_ was eluted at 8.566 min. **e** pH responsiveness of imidized DBCO-PEG. Imidized DBCO-PEG was treated with PBS (pH 5.0, black; pH 6.5, red; pH 7.4, blue) for varying time points. At the fixed time-point, the content of DBCO-NH_2_ was analyzed by HPLC to reflect the hydrolytic efficiency of imidized DBCO-PEG. Data are represented as the mean ± standard deviation (n=3). The two-way analysis of variance (ANOVA) was used to determine the significance among groups.


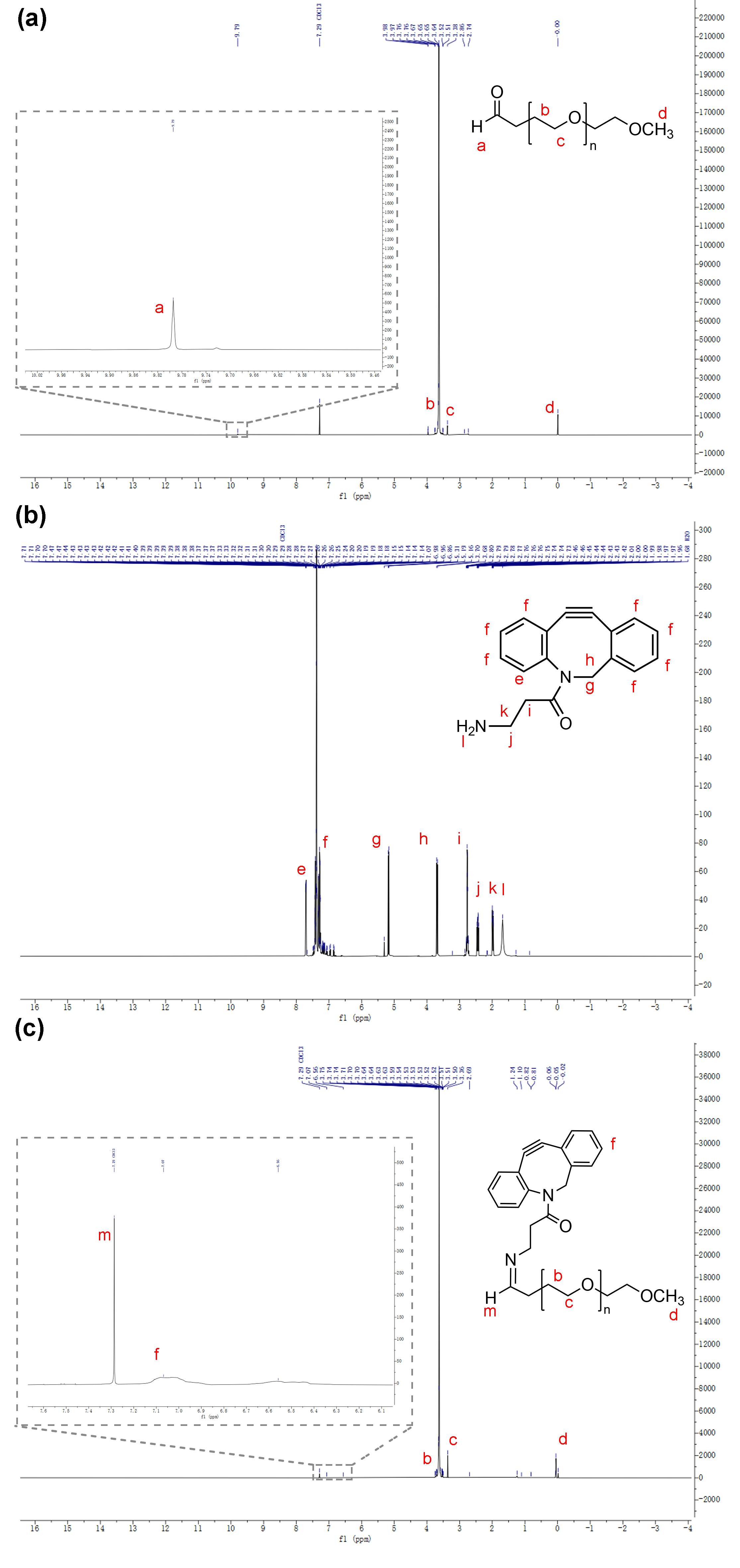


**Fig. S17** ^1^H NMR spectra of mPEG-CHO (**a**), DBCO-NH_2_ (**b**) and imidized DBCO-PEG (**c**). Characteristic peaks (a-m) for each sample were assigned to its chemical structure, as labeled on the spectra.


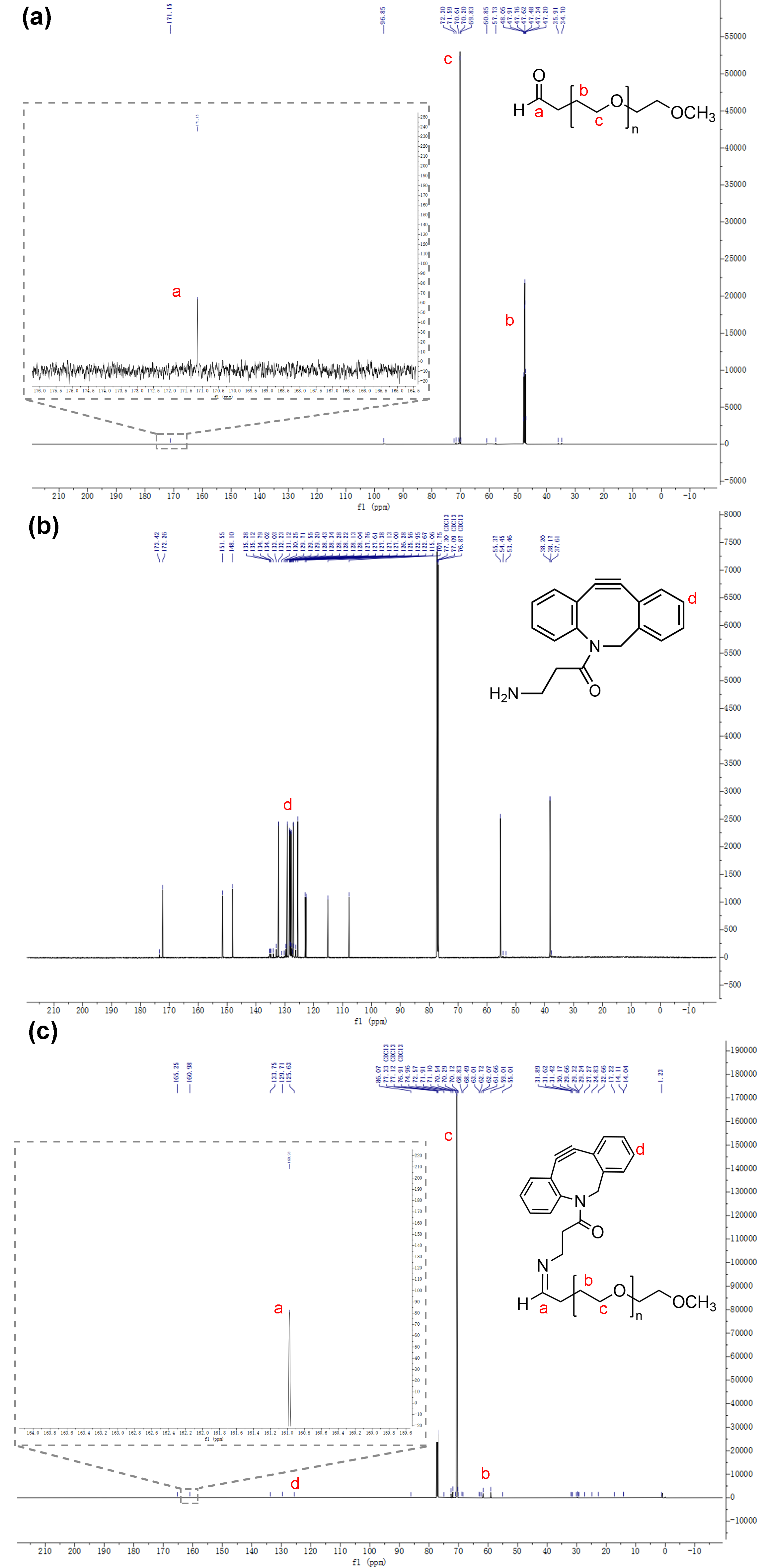


**Fig. S18** ^13^C NMR spectra of mPEG-CHO (**a**), DBCO-NH_2_ (**b**) and imidized DBCO-PEG (**c**). Each sample’s characteristic peaks (a-d) were assigned to its chemical structure, as labeled on the spectra.

**Table S1 Plasmid sequences in Dataset S1**

| **ID** | **Name** | **Description** |
| --- | --- | --- |
| 1 | pCMV-VSV-G-D192 | Plasmid encoding mProtein D192 |
| 2 | pCMV-VSV-G-A246 | Plasmid encoding mProtein A246 |
| 3 | pCMV-VSV-G-A247 | Plasmid encoding mProtein A247 |
| 4 | pCMV-capsid10-flag | Plasmid encoding capsid 10 |
| 5 | pCMV-cargoGFP | Plasmid encoding cargoGFP |
| 6 | pCMV-cargoCas9 | Plasmid encoding cargoCas9 |
| 7 | pCMV-cargoshTNFα | Plasmid encoding cargoshTNFα |
| 8 | pCMV-cargoshcontrol | Plasmid encoding cargoshcontrol |
| 9 | pLKO-sgRNA | Plasmid encoding sgRNA for αE*_myc_* |

**Table S2 Oligos used in this study**

| **Name** | **Sequence (5'-3')** | **Descriptions** |
| --- | --- | --- |
| quantification FP | CCGATACACGCGTTTCCAAC | quantification primers for 5' UTR fragment in capsid 10 |
| quantification RP | CTCGTGGTTGGCGTCTTTTG |  |
| sgRNA | GAAGGAGAAAGTCAGTCCGC | sgRNA for αE*_myc_* |
| genome PCR FP | TGGAGGCACAGCCTGAAGGC | genome PCR |
| genome PCR RP | CAGCCTTCTAGCCATCCTCT |  |
| shTNFα | AAGTGCCTATGTCTCAGCCTCTT | shRNA sequences |
| shcontrol | TTCTCCGAACGTGTCACGT |  |
| Myc FP | GCCACGTCTCCACACATCAG | primers for qRT-PCR |
| Myc RP | TCTTGGCAGCAGGATAGTCCT |  |
| GAPDH FP | ACTTTGGTATCGTGGAAGGACT |  |
| GAPDH RP | GTAGAGGCAGGGATGATGTTCT |  |

**Dataset S1 (separate file)**

Files of plasmid sequences.

**Dataset S2 (separate file)**

Quantification template for gene cargoes packaged in biosome.

**SI References**

1. J. Wang, R. M. Wolf, J. W. Caldwell, P. A. Kollman, D. A. Case. Development and testing of a general amber force field. J Comput Chem. 25(9), 1157-1174 (2004). https://doi.org/10.1002/jcc.20035

2. D. A. Pearlman, D. A. Case, J. W. Caldwell, W. S. Ross, T. E. Cheatham, S. Debolt, D. Ferguson, G. Seibel, P. Kollman. Amber, a package of computer-programs for applying molecular mechanics, normal-mode analysis, molecular-dynamics and free-energy calculations to simulate the structural and energetic properties of molecules. Comput Phys Commun. 91(1-3), 1-41 (1995). https://doi.org/10.1016/0010-4655(95)00041-D

3. W. L. Jorgensen, J. Chandrasekhar, J. D. Madura, R. W. Impey, M. L. Klein. Comparison of simple potential functions for simulating liquid water. J Chem Phys. 79(2), 926-935 (1983). https://doi.org/10.1063/1.445869

4. R. Luo, L. David, M. K. Gilson. Accelerated poisson-boltzmann calculations for static and dynamic systems. J Comput Chem. 23(13), 1244-1253 (2002). https://doi.org/10.1002/jcc.10120

5. M. L. Connolly. Solvent-accessible surfaces of proteins and nucleic acids. Science. 221(4612), 709-713 (1983). https://doi.org/10.1126/science.6879170

6. M. L. Connolly. Analytical molecular surface calculation. J Appl Crystallogr. 16(5), 548-558 (1983). https://doi.org/10.1107/S0021889883010985

7. C. Bao, J. Duan, Y. Xie, Y. Liu, P. Li, J. Li, H. Zhao, H. Guo, Y. Men, Y. Ren, J. Xu, G. Wang, W. Lu. A novel oncogenic enhancer of estrogen receptor-positive breast cancer. Mol Ther - Nucl Acids. 29, 836-851 (2022). https://doi.org/10.1016/j.omtn.2022.08.029

8. H. Wang, S. A. Marsters, T. Baker, B. Chan, W. P. Lee, L. Fu, D. Tumas, M. Yan, V. M. Dixit, A. Ashkenazi, I. S. Grewal. Taci-ligand interactions are required for t cell activation and collagen-induced arthritis in mice. Nat Immunol. 2(7), 632-637 (2001). https://doi.org/10.1038/89782
